# Future rice farming threatened by drought in the Lower Mekong Basin

**DOI:** 10.1038/s41598-021-88405-2

**Published:** 2021-04-30

**Authors:** Hyunwoo Kang, Venkataramana Sridhar, Mohammed Mainuddin, Le Duc Trung

**Affiliations:** 1 Department of Biological Systems Engineering, Virginia Tech, Blacksburg, VA 24061 USA; 2 Water Security Program|CSIRO Land and Water, Black Mountain Laboratories, GPO Box 1700, Canberra, ACT 2601 Australia; 3 Viet Nam National Mekong Committee, 23 Hang Tre, Hanoi, Vietnam

**Keywords:** Hydrology, Climate change

## Abstract

The Lower Mekong River basin (LMB) has experienced droughts in recent decades, causing detrimental economic losses and food security conundrums. This study quantified the impact of climate change on drought, and rainfed rice production in the LMB. The Soil and Water Assessment Tool (SWAT) and AquaCrop models were used to evaluate long-term drought indices and rainfed rice yields under historical and future climate conditions (1954–2099) with four climate models and two emission scenarios (RCP 4.5 and RCP8.5) from the Coupled Model Intercomparison Project Phase 5 (CMIP5). We found that rice yield might increase (24–43%) due to the elevated levels of atmospheric CO_2_ concentration (+ 34.3 to + 121.9%) and increases in precipitation. Contrastingly, considerable decreases in rice yield up to 1.5 ton/ha in the Vietnam Central High Plain (VCHP) region could be expected resulting from reduced precipitation by about 34% during drought years. To avert any major food crisis, an expansion of irrigation areas could be required to compensate for the expected reduction in rice yields. We conclude that a framework combining hydrology and crop models to assess climate change impacts on food production is key to develop adaptation strategies in the future.

## Introduction

Drought is a natural disaster that has a detrimental influence on water resources, economy, environment, and crop production^1–3^. Drought has affected many regions around the world over the last few decades^4,5^, and it has been exacerbated by climate change^6–8^. Besides, drought has a particularly adverse impact on crop production as well as regional and global food security^9^.

The Lower Mekong Basin (LMB) contains parts of the countries of Cambodia, Lao PDR, Thailand, and Vietnam, where the countries have vulnerable populations exacerbated by poverty and food insecurity^10^. The LMB has experienced some severe droughts in the past three decades^11–13^, and these droughts inflicted significant crop losses, reductions in fishery and livestock yields, harmful levels of salinity intrusion, and widespread shortage for domestic and industrial water use^11,14^. Notably, droughts in 1991–1994 and 2015–2016 were the longest and driest droughts, respectively^12,15^. The 2015–2016 drought brought serious economic losses to Thailand (1.7 billion USD^14^), and more than 9.56 million people were affected. In the Mekong Delta region, the worst saline intrusion was recorded^16^, and 30% of winter and spring crops were threatened^17,18^.

A week monsoon during the flood season and a warm ENSO phase (El Niño/Southern Oscillation) have derived severe droughts and significant restrictions on rice production in the LMB^13,19^, and the severity of droughts would be exacerbated by climate change, specifically in the Lower LMB and 3S regions (Sekong, Sesan and Srepok)^20^. The LMB is one of the most crucial regions for rice production, which is not only providing food for more than 70 million inhabitants^21^ but also contribute to global food security^22^. The average total rice production in LMB during 2009–2013 was 52.5 million tons, and average yields for Cambodia, Lao PDR, Thailand, and Vietnam were 3.0, 3.2, 2.5, and 5.2 ton/ha, respectively^23,24^. Thailand and Vietnam belong to the five major rice exporters of the world in 2017^25^, and the Mekong Delta in Vietnam and Khorat Plateau in Thailand produce about 75% of all rice production in the LMB^21^. Rice productions from the LMB are required to increase up to 50% in the next 30 years (about 80 million tons) to meet the demand for a growing population^21,26^. Thus, decreases in rice production due to failed monsoon, climate change, and drought in the LMB will adversely impact the regional to global food security^27^.

Numerous studies have evaluated the impacts of climate change on rice yield in the LMB. Kontgis et al.^28^ simulated the crop environment resources synthesis rice model (CERES-Rice) within the decision support system for agrotechnology transfer (DSSAT) platform^29^. This study compared rice yields with and without the impacts of the irrigation and CO_2_ fertilization effect, the latter of which is attributed to an increased rate of photosynthesis in plants as a result from increased CO_2_ levels^30^. Furthermore, these results pointed out that there were decreases in rice yields in the middle of twenty-first century without the irrigation and CO_2_ fertilization effect in the Mekong Delta. However, considering CO_2_ fertilization effect of RCP 4.5 and 8.5 emissions, increases in rice yield up to 23% were plausible, and that could compensate for yield declines by increasing temperatures^28^. Jiang et al.^31^ assessed the impacts of climate change on rice production in the Mekong Delta using DSSAT and global climate models. The results implied that reductions of rainfed rice occurred due to increasing temperatures and seasonal variations of precipitation, but the elevated CO_2_ resulted in remarkable increases in rice productions. Chun et al.^32^ demonstrated that improved irrigation strategy and CO_2_ fertilization effect could cause significant increases in rice yields (up to 8.2–42.7%) in the 2080s under RCP 8.5 in Cambodia and Thailand. Poulton et al.^33^ found that CO_2_ level and rainfall variability have significant influences on rainfed rice production in Cambodia after 2030. Also, they concluded that expanding irrigation areas would be required to compensate for the impacts of climate change.

Thus far, several studies concluded that a combination of CO_2_ fertilization and irrigation strategies may lead to increases in rice production or at least compensate for the yield losses due to the impacts of climate change in the LMB. However, other studies showed different results in the regional scale analysis. Boonwichai et al.^34^ simulated rainfed rice using the DSSAT crop simulation model in the Songkhram River Basin of Thailand, and rice yields reduced by 14% and 10% under the RCP4.5 and RCP8.5, respectively. The reductions in rice yields were mainly due to the increases in temperature and crop water use. Only a few studies demonstrated the impacts of climate change and resulting drought on rice yields, including for the LMB region where a significant cultivable land area was used for rice cultivation. Trisurat et al.^35^ used the InVEST model (Integrated Valuation of Ecosystem Services and Tradeoffs) and estimated rice production in the LMB. They found that rice production would decrease up to 4.2% due to the extreme drought conditions, and these reductions were mostly confined to Thailand and Lao PDR. However, the lack of an integrated assessment using a physically-based hydrologic model, climate models, and crop simulation is evident for evaluating future drought and rice yield in rainfed areas^36^. This approach is essential to quantify the impacts of droughts and rice yield at a scale appropriate for decision making by explicitly accounting for increased biomass production from the CO_2_ fertilization effect. It is also imperative to evaluate *what-if* scenarios for expanding irrigated areas to mitigate any crop loss due to drought and to avoid potential reductions in rice yield.

The objectives of this study are to evaluate drought and rice yield in the LMB at the provincial scale over the historical period (1956–2019) and to assess the impacts of drought on rainfed rice yield in the next 80 years (2020–2099) with two future climate projection periods (f1: 2020–2059, f2: 2060–2099). This study provides crop yield under future drought scenarios using soil moisture-based drought index, crop simulation model, and high-resolution meteorological dataset, and the results of this transnational study can serve as a viable guideline for planning water resources and land management for alleviating food security concerns in the face of natural and human-induced changes in LMB.

## Methods

**Figure 1:**
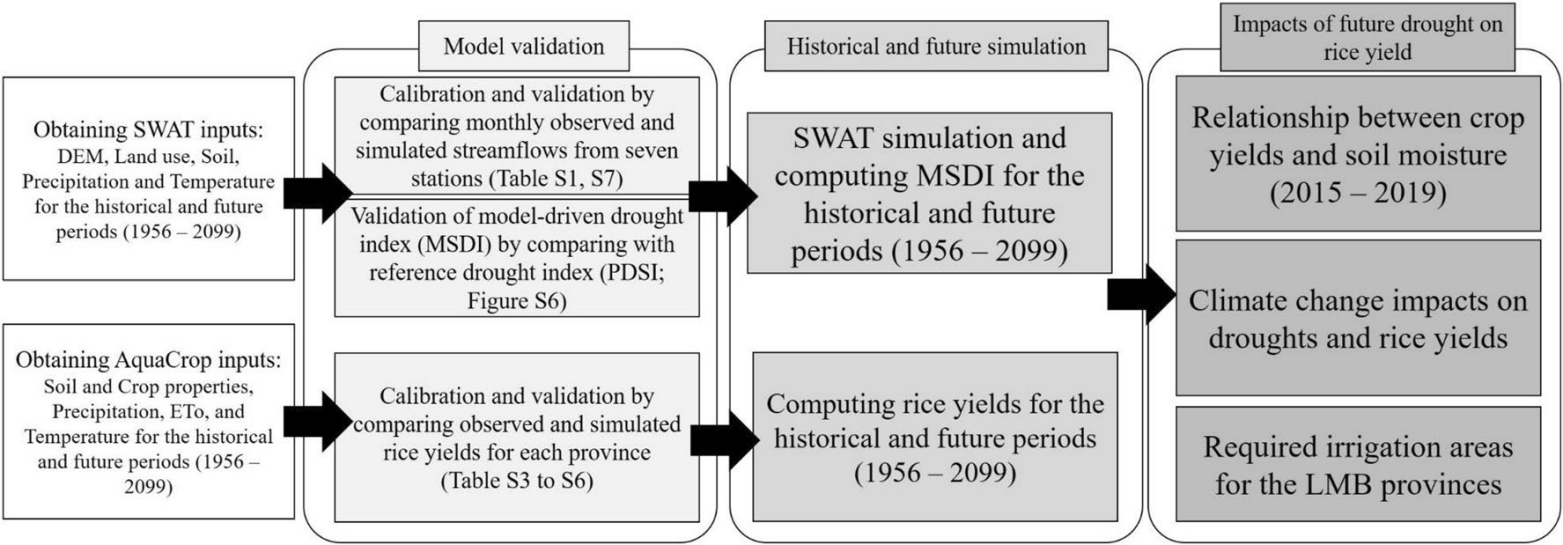
The process flow diagram showing hydrological and crop simulations. *SWAT* Soil and Water Assessment Tool, *MSDI* Multivariate Standardized Drought Index, *PDSI* Palmer Drought Severity Index.

Figure 1 outlined the overall approaches and methods that were used in this study. First, input data required for the Soil and Water Assessment Tool (SWAT)^37^ and AquaCrop^38^ models for the LMB were obtained. Second, the SWAT model was calibrated by comparing monthly streamflows from seven stations, and the AquaCrop model was calibrated using the observed rice yields for each province. Third, SWAT-driven soil moisture was used to compute a drought index for the historical and future periods. Forth, AquaCrop was used to estimate rice yields for the historical and future periods. Following this, the climate change impacts on droughts, rice productions, and required irrigation areas were evaluated based on the drought index and rice yield simulations.

### Study area

**Figure 2:**
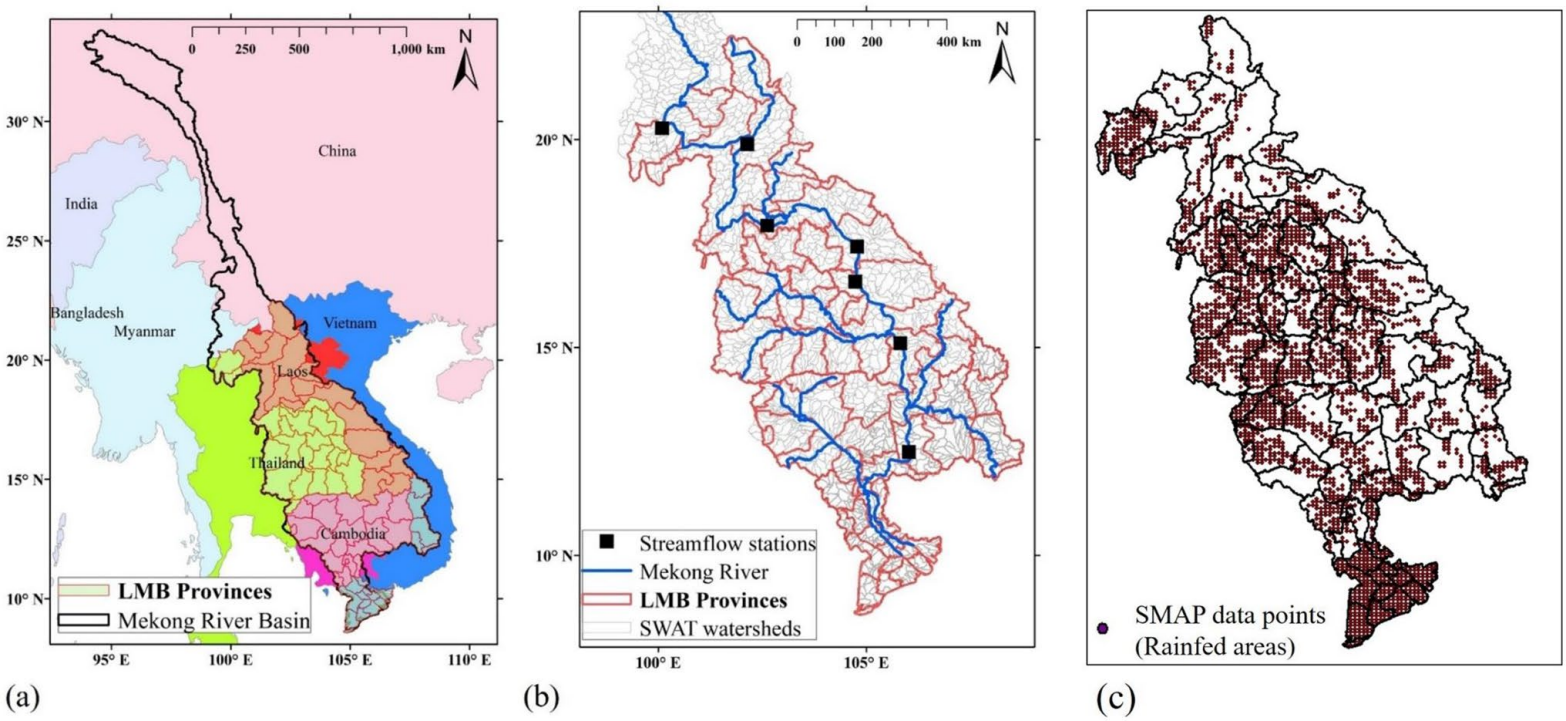
Study area. (**a**) Spatial map of the entire Mekong River Basin and the Lower Mekong Basin (LMB). (**b**) Spatial map of the LMB. Red lines indicate the boundary of provinces, blue lines represent the Mekong River, grey lines are the delineated SWAT sub-watersheds, and black and square boxes are streamflow stations for the SWAT calibration. (**c**) Soil Moisture Active Passive (SMAP) data points. Brown points indicate the SMAP data points where are intersected with the rainfed areas (3065 points). Spatial maps were created using ArcMAP10.5 software by Esri (www.esri.com).

This study focused on the entire LMB. It covers an area of 642,000 km^2^, roughly consisting of 76% of the Mekong River Basin (Fig. 2a). The river originates in China and flows about 2300 kms to reach near Chiang Saen and this segment is called the upper Mekong River. Below this point, the river becomes the Lower Mekong River which flows for about 2600 km. The lower segment of the river passes through uplands of Laos, Cambodia and Vietnam, and forms the delta near the mouth where it drains into the South China Sea^21^. The LMB is influenced by the tropical monsoon, which generates distinct wet and dry seasons; the wet season is from May to October, and the dry season is from November to April. Several major drought events have hit the region over the last decades, and they generated detrimental impacts on water resources, rice cultivations, livelihood activities, and food security^11,12,15^. For this investigation, we have focused on the rainfed rice yield analysis because it is a primary crop in the LMB, and it accounts for 58% of the paddy area of the LMB^21^. Besides, rainfed areas are vulnerable in a changing climate due to precipitation variability than irrigated areas^39^, and a better irrigation management system which can minimize the adverse effects of drought in the LMB^40^.

### SWAT model and inputs

The SWAT model^37^ is a semi-distributed and river basin scale model, which is designed to simulate watershed responses under various climatic and geologic conditions. SWAT has been widely applied for the evaluation of diverse aspects of drought, such as historic drought assessments^7,8,10,41^, drought forecasting^42,43^, and climate change impacts on droughts^6–8,39^. The SWAT model requires a digital elevation model (DEM), soil data, land use, and weather dataset (e.g., daily precipitation and maximum and minimum temperature). The Global Multi-resolution Terrain Elevation Data 2010 (GMTED2010; 250 m resolution)^44^, soil data obtained from the soil dataset of the Food and Agriculture Organization of the United Nations^45^, and the Global Land Cover Characterization (GLCC) to estimate the land use parameters were used as inputs for SWAT simulations^46^. For the historical period (1951–2019), a 0.25-degree resolution of precipitation and temperature data were used^47–49^. Sridhar et al.^50^ developed model inputs and calibration parameters for the Mekong River Basin, which will be used in this analysis (Supplementary Table S1 in the Supplementary Information).

### AquaCrop model and inputs

AquaCrop is a crop growth model developed by the Land and Water Division of the Food and Agricultural Organization (FAO), and it is a process-based model that considers crop growth, atmospheric, soil, crop, and management conditions^38,51^. AquaCrop requires a relatively small number of inputs and parameters, and it simulates yield response to water of crops and is suited to explain conditions where water is a crucial factor in crop production^38^. AquaCrop has been widely used for investigating climate change impacts on crop production and food security in many regions in the world, particularly in the LMB^3,52^. Biomass production and yield formation in AquaCrop were based on Eqs. (1) and (2) ^38^.

1 $$B={WP}^{*}\sum \left(\frac{{Tr}_{i}}{{ETo}_{i}}\right),$$

2 $$Y=HI\times B,$$

where B is the cumulative biomass production (ton/ha), WP^*^ is the biomass water productivity normalized for climate, Tr_i_ is the daily crop transpiration (mm/day), ETo_i_ is the daily reference evapotranspiration (mm/day), Y is the final crop yield (ton/ha), and HI is the harvest index. Raes et al.^38^ pointed out that an increased CO_2_ level, which induces CO_2_ fertilization, derives slight decreases in the crop transpiration (Tr) and significant increases in the biomass water productivity (WP^*^) for the AquaCrop simulation. Besides, a temperature is likely to increase the evaporating power of the atmosphere (ETo).

AquaCrop requires soil properties, daily precipitation, minimum and maximum temperature, reference evapotranspiration (ETo), planting date, and the growing period, which are relatively a few parameters and inputs to simulate the crop yield in response to various water availability conditions^38,40^. For the historical and future simulations, soil properties were extracted from the FAO soil dataset^44^. Forcing datasets include daily precipitation, minimum and maximum temperature data and the same inputs used for SWAT simulations were again utilized after aggregating them for each province. Besides, other variables namely reference evapotranspiration (ETo) and CO_2_ concentration for each RCP were derived from SWAT as PET and Nazarenko et al.^53^, respectively.

### Model-driven drought index

SWAT simulated soil moisture was used to compute the multivariate standardized drought index (MSDI)^54^. MSDI is based on the joint distribution and probability of two hydro-meteorological variables such as precipitation and soil moisture. For this study, monthly soil moisture estimations of entire soil columns (about 2 m) from the SWAT model and precipitation data were used to evaluate historic and future drought conditions in the 74 provinces in the LMB.

The joint distribution of two variables (X and Y) is defined as Eq. (3):

3 $$P\left(X\le x, Y\le \mathrm{y}\right)=p,$$

where *p* is joint probability of two variables, and MSDI is described as Eq. (4):

4 $$MSDI={\varnothing }^{-1}\left(p\right),$$

where $\varnothing$ is the standard normal distribution.

For the MSDI estimation, the Gringorten plotting position formulas is used to reduce the uncertainties in fitting parameters^55^, and the joint distribution was computed by Eq. (5);

5 $$P\left({x}_{k}, {y}_{k}\right)=\frac{{m}_{k} -0.44}{n +0.12},$$

where $$n$$ is the number of observations, and $${m}_{k}$$ is the number of occurrences when the pair ($${x}_{i},{y}_{i})$$ is $${{x}_{i}\le x}_{k}$$ and $${{y}_{i}\le y}_{k}$$. Supplementary Table S2 presents drought classifications of MSDI. The negative values (MSDI < − 1.0) indicate the drought conditions, while positive values represent the wet conditions.

### Model calibration

The SWAT model calibration was performed by comparing monthly streamflows at seven stations (Fig. 2b; Supplementary Table S3), and the SUFI-2 algorithm in the SWAT calibration and uncertainty assessment tool (SWAT-CUP)^56^ was used. The model evaluation was performed based on the Nash–Sutcliffe coefficient (NS)^57^ (Eq. 6).

6 $$NS=\frac{\sum_{i=1}^{n}{\left({Q}_{s}^{i}-{Q}_{o}^{i}\right)}^{2}}{\sum_{i=1}^{n}{\left({Q}_{o}^{i}-\stackrel{-}{{Q}_{o}}\right)}^{2}},$$

where $\stackrel{-}{{Q}_{o}}$ is the mean of observed discharge, ${Q}_{s}$ is simulated discharge, ${Q}_{o}^{i}$ is observed discharge at time *i.* In addition, the model-driven MSDI was validated by the comparisons with a reference drought index (Palmer Drought Severity Index; PDSI) at a moderate drought category (PDSI < − 2, MSDI < − 1). Supplementary Figure S1 presents the selected locations for drought assessments, and they were highlighted as red points for PDSI and black areas for MSDI.

The model accuracy of the AquaCrop model was assessed by comparing observed and simulated rice yields for each province, and the performance was verified by calculating the root mean square error (RMSE) (Eq. 6), which has been widely used for crop simulation studies^3,40,58^. For the baseline simulations, planting date, harvest index (HI), and fertilizer stress were used as the calibration parameters, and they have been applied for the AquaCrop simulations (Supplementary Tables S4–S7 in the Supplementary Information)^3,54,59^. Based on the assessment of root mean square error (RMSE), the simulation was found to be satisfactory for the LMB region, and the detailed discussion is provided in the later sections.

7 $$\mathrm{RMSE}= \sqrt{\frac{\sum_{i=1}^{N}{\left({Simulated}_{i}-{Observed}_{i}\right)}^{2}}{N}}\left(\mathrm{Unit}:\frac{\mathrm{t}}{\mathrm{ha}}\right),$$

where, *simulated*_*i*_ and *observed*_*i*_ are the simulated and observed rice yield, and N is the number of observations. The performance of the simulation improves as RMSE approaches zero.

### Satellite-based soil moisture and rice yield

Soil moisture is an essential variable for monitoring agricultural production and drought, and it is appropriate to evaluate the impact of water deficit^54,60^. In particular, the regions of rainfed agriculture are largely affected by soil moisture deficits during the crop growing season^61^. In this study, the simulated rice yields and droughts were compared with a satellite-based root zone soil moisture observation from Soil Moisture Active Passive (SMAP, Level 4)^62^ to understand the relationship between rice yield, precipitation, and drought conditions. SMAP provides three hourly soil moisture data with a 9 km spatial resolution from April 2015, and 3065 grids correspond to the rainfed areas in the LMB (Fig. 2c).

### Impacts of drought on rice yield

In this study, the crop yield analysis for the rainfed rice was performed at the provincial scale using the observed agricultural production data in the LMB (Fig. 2b). Observed crop yield data was obtained from the official statistical websites or reports of the countries in the LMB^24,40^. The study area contains 74 provinces in the LMB, and there are 20, 17, 22, and 15 provinces in Cambodia, Loa PDR, Thailand, and Vietnam, respectively. The average area of all the 74 provinces is 8079 km^2^, the largest province is Savannakhet in Lao PDR (21,407 km^2^), and the smallest is Phnom Penh in Cambodia (373 km^2^). However, the Vietnam Central High Plain (VCHP) region does not include the provinces entirely because the current boundaries are based on the Mekong River basin, not on a provincial border (Fig. 2).

To figure out the impacts of drought on rainfed rice, we divided drought and non-drought years and compared the rice yields. The drought and non-drought years were separated whether the MSDI values were below average or not. To mitigate the impacts of drought, rainfed areas would need irrigation as they serve as a shock absorbed during the times of failed monsoon. While the expanded irrigation shields the farmers from facing the losses in crop yield, it can potentially alleviate food security and livelihood concerns. The required irrigation area (RIA) was calculated to quantify how much additional irrigation areas would be needed to compensate for the yield losses during the drought years, and RIA was calculated by Eqs. (8) and (9).

8 $$YL=\left({Y}_{n}-{Y}_{d}\right)\times {A}_{rice,}$$

9 $$RIA=\frac{YL}{{Y}_{n}},$$

where, $YL$ is the yield losses (ton), ${Y}_{n}$ is the rice yield during the non-drought years (ton/ha), ${Y}_{d}$ is the rice yield during the drought years (ton/ha), ${A}_{rice}$ is the rice cultivated area for each province (ha), and $RIA$ is the required irrigation area (ha).

### Climate projections

The Coupled Model Intercomparison Project Phase 5 (CMIP5) climate model outputs are widely used to assess historical and future climate impacts with higher reliability in the projected precipitation and temperature. In this study, four CMIP5 climate models, GFDL-ESM2M, IPSL-CM5A-LR, MIROC-ESM-CHEM, and NorESM1-M were implemented with two representative concentration pathways (RCP4.5 and RCP8.5), and they captured a wide range of precipitation (+ 18.3 to + 47.0%) and temperature changes (+ 1.1 to + 4.6 °C) in the LMB. We directly used statistically downscaled and bias-corrected data at 0.25-degree resolution that were available by the intersectoral impact model intercomparison project (ISI-MIP)^63^.

**Figure 3:**
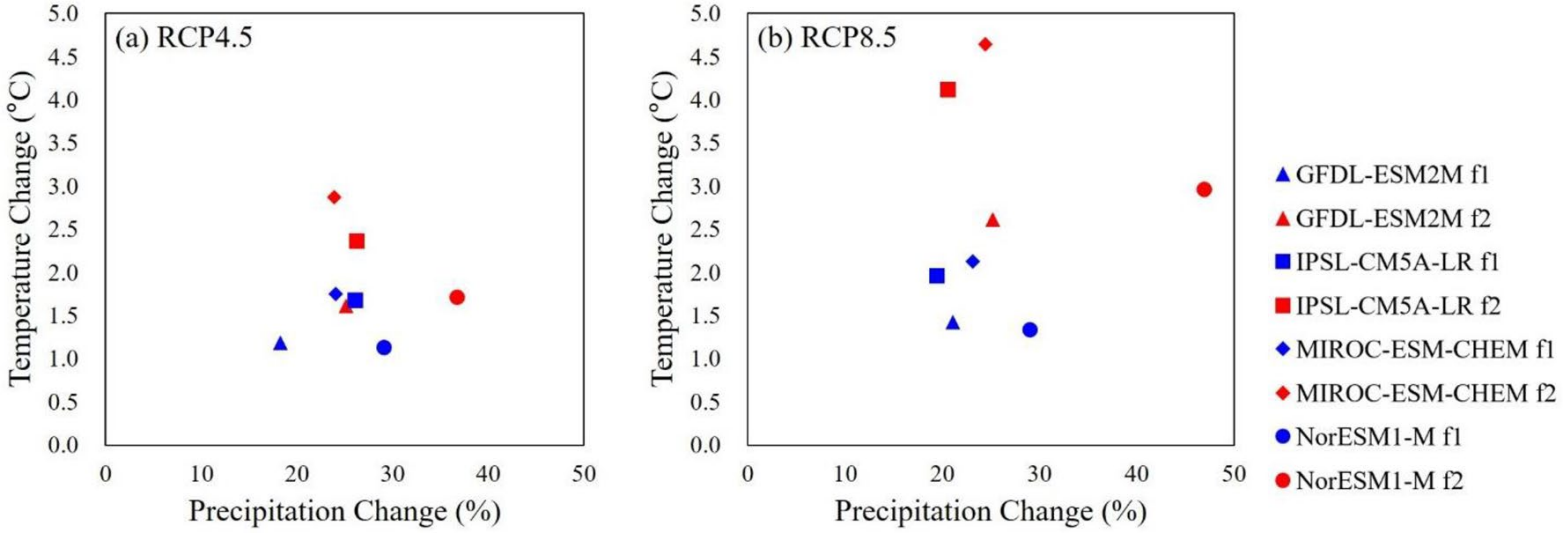
Precipitation and temperature change in the LMB. The X-axis is the change in precipitation (%), and the Y-axis is the change in temperature (°C). All of the dots indicate the change in precipitation and temperature for each climate model and period. The blue dots represent the f1 period, and the red dots indicate the f2 period. (**a**) RCP 4.5. (**b**) RCP 8.5.

As temperature and precipitation characterizations are needed for drought and crop growth modeling, especially under rainfed conditions, the evaluation of precipitation and temperature for their trends and future projections are deemed necessary. Figure 3 presents changes in mean values of precipitation and temperature for the four GCMs, RCPs, and future periods. Among the climate models and periods, GFDL-ESM2M and RCP4.5—f1 period was the driest model that showed the lowest precipitation increase (18.3%), while NorESM1-M and RCP8.5—f2 period was the wettest model, which presented the highest precipitation increase (47.0%). In addition, NorESM1-M and RCP4.5—f1 period was a cold model that showed the lowest temperature increase (1.1 °C), while MIROC-ESM-CHEM and RCP8.5 f2 was a hot model, which presented the highest temperature increase (4.6 °C). Supplementary Figure S2 in the Supplementary Information shows the spatial maps of historical annual precipitation and future changes for each climate model and period, and Supplementary Fig. S2a,b are the results of RCP4.5 and RCP8.5, respectively. For the historical period, annual precipitation ranged from 933 to 2070 mm. For the future periods, the means of four climate models in the entire LMB indicated that there were 24.7–28.0% increases for RCP4.5, and 23.2–29.3% increases for RCP8.5 in the f1 and f2 periods, respectively. It is clear that the overall pattern of increased but mostly heterogeneous precipitation in the future that was higher than the present periods could impact drought and crop yield. Supplementary Figure S3 presents historical average temperature and future changes for the entire LMB. For the historical period, the average temperature ranged from 21.3 to 28.8 °C. For the future periods, there were overall temperature increases for the entire LMB. Temperature increases ranged from 0.29 to 3.75 °C for RCP4.5, and 0.54–5.71 °C for RCP8.5.

Although the overall precipitation increases were expected, a significant reduction in precipitation during the crop growing season as projected could cause a yield reduction. Tables 1 and 2 show the total amount of precipitation during crop growing season for the historical and future periods and precipitation differences between drought and non-drought years for future periods. Table 1 present the mean values of four GCMs, and Table 2 shows the precipitation differences for each climate model and the future period. In Table 1, the numbers below the precipitation amount indicate the number of droughts and non-drought years, and they are approximately 30 years that are half of the historical period. However, there were decreases in a number of drought years and increases in non-drought years for the future periods, which were particularly noticeable in the results of a climate model with high precipitation increases (e.g., NorESM1-RCP4.5 model and f2 period). In the following sections, this assessment serves as the basis for evaluating the effect of temperature and precipitation changes on drought and crop yield in the rainfed agriculture areas of the basin.

**Table 1:** Total precipitation amount during crop growing season for the historic and future periods and differences between drought and non-drought year (Average of four GCMs).

				RCP4.5						RCP8.5					
Periods	Historical (1956–2015)			f1 (2020–2059)			f2 (2060–2099)			f1 (2020–2059)			f2 (2060–2099)		
Countries	Precipitation (mm)		Precipitation difference (%)	Precipitation (mm)		Precipitation difference (%)	Precipitation (mm)		Precipitation difference (%)	Precipitation (mm)		Precipitation difference (%)	Precipitation (mm)		Precipitation difference (%)
	DY	NDY		DY	NDY		DY	NDY		DY	NDY		DY	NDY	
Cambodia	823 (36)	847 (24)	− 2.9	1088 (15.8)	1,096 (24.2)	− 1.0	1128 (17.5)	1107 (22.5)	1.6	1075 (18.2)	1090 (21.8)	− 1.6	1109 (16)	1121 (24)	− 1.2
Lao PDR	1130 (31)	1195 (29)	− 5.8	1347 (21)	1462 (19)	− 8.6	1386 (19)	1509 (21)	− 8.9	1301 (20.8)	1477 (19.2)	− 13.6	1393 (19.8)	1532 (20.2)	− 10.1
Thailand	786 (36)	846 (24)	− 7.7	960 (16.2)	1006 (23.8)	− 5.0	1019 (15.5)	1061 (24.5)	− 4.3	945 (16)	1027 (24)	− 8.7	1018 (18.5)	1064 (21.5)	− 4.7
VCHP	831 (31)	1014 (29)	− 22.1	1061 (13.2)	1286 (26.8)	− 21.5	1062 (15)	1222 (25)	− 15.3	1006 (14)	1245 (26)	− 23.5	1034 (14.5)	1281 (25.5)	− 23.6
Mekong Delta	888 (32)	941 (28)	− 6.1	1142 (17.5)	1114 (22.5)	2.0	152 (19.5)	1155 (20.5)	− 0.3	1158 (18.8)	1138 (21.2)	1.7	1189 (18.2)	1186 (21.8)	0.2

The numbers in the bracket indicate the average number of drought and non-drought years for four GCMs. Negative values indicate the decreases in precipitation during drought years.

*DY* Drought year, *NDY* Non-drought year, *VCHP* Vietnam Central High Plain.

**Table 2:** Precipitation differences during crop growing season between drought and non-drought year for each climate model and future period (Unit: %).

RCPs	Countries	GFDL-ESM2M		IPSL-CM5A-LR		MIROC-ESM-CHEM		NorESM1-M	
		f1 (%)	f2 (%)	f1 (%)	f2 (%)	f1 (%)	f2 (%)	f1 (%)	f2 (%)
RCP4.5	Cambodia	7.3	7.7	1.7	2.0	− 4.8	− 2.4	− 8.2	− 0.8
	DY-NDY	19–21	17–23	17–23	21–19	18–22	22–18	9–31	10–30
	Lao PDR	− 11.4	− 3.7	− 8.0	− 10.5	− 11.6	− 10.0	− 3.4	− 11.5
	DY-NDY	22–18	21–19	16–24	25–15	26–14	20–20	20–20	10–30
	Thailand	− 6.7	3.4	4.6	− 4.4	− 13.9	− 8.7	− 4.1	− 7.5
	DY-NDY	16–24	16–24	13–27	21–19	20–20	17–23	16–24	8–32
	VCHP	− 15.4	− 12.0	− 11.9	− 6.4	− 34.4	− 28.4	− 24.4	− 14.4
	DY-NDY	16–24	15–25	12–28	18–22	12–28	17–23	13–27	10–30
	Mekong delta	16.5	3.4	− 9.7	− 3.0	− 0.2	− 1.1	1.6	− 0.7
	DY-NDY	16–24	23–17	20–20	18–22	14–26	21–19	20–20	16–24
RCP8.5	Cambodia	4.4	− 1.7	− 0.6	2.0	− 9.7	− 4.1	− 0.5	− 1.0
	DY-NDY	18–22	19–22	18–22	20–20	19–21	19–21	18–22	6–34
	Lao PDR	− 16.7	− 9.4	− 15.3	− 13.3	− 7.0	− 10.4	− 15.7	− 7.2
	DY-NDY	22–18	22–18	16–24	24–16	25–15	19–21	20–20	14–26
	Thailand	− 2.4	− 9.3	− 13.7	− 5.4	− 12.5	− 3.5	− 6.2	− 0.8
	DY-NDY	19–21	23–17	14–26	21–19	15–25	16–24	16–24	14–26
	VCHP	− 25.5	− 24.8	− 17.0	− 13.6	− 29.8	− 25.9	− 22.7	− 29.9
	DY-NDY	16–24	11–29	12–28	18–22	15–25	22–18	13–27	7–33
	Mekong delta	− 0.1	2.9	7.5	0.4	− 2.4	− 1.1	1.7	− 1.6
	DY-NDY	18–22	21–19	21–19	18–22	14–26	21–19	22–18	13–27

The numbers of drought and non-drought year were stated below the differences. Negative values indicate the decreases in precipitation during drought years.

*DY* Drought year, *NDY* Non-drought year, *VCHP* Vietnam Central High Plain.

## Results and discussion

### SWAT and drought evaluations

The SWAT model was calibrated using SWAT-CUP^56^ and the assessment was carried out by comparing monthly streamflows from seven stations. Supplementary Table S3 in the Supplementary Information presents the values of R^2^ and NS efficiency, and all seven stations that showed values above 0.8, are considered ‘Very good’ for the monthly simulation^64^.

Long-term historical drought conditions (1954–2014) were also validated with Palmer Drought Severity Index (PDSI)^65^ and multivariate standardized drought index (MSDI) at a moderate drought category (PDSI < − 2, MSDI < − 1) (Supplementary Table S2). Supplementary Figure S1 in the Supplementary Information shows the locations of PDSI and SWAT sub-watersheds, and the numbers in the yellow boxes correspond to specified points on the map. The model-driven MSDI generated 4.0, 10.1, and 15.9% of extreme, severe, and moderate droughts, and they captured between 56 and 73% of the drought occurrences in this period. Factors responsible for the discrepancies in these comparisons were manifold. First, the PDSI data was at 2.5-degree resolution, while the estimated MSDI was at 0.25-degree, primarily driven by the resolution of meteorological data. Second, the two different sources of meteorological data were applied for this assessment. For example, PDSI was calculated by the precipitation data from the National Centers for Environmental Prediction (NCEP) Climate Prediction Center (CPC)^66^, while the model-based MDSI used the other precipitation data^47^_,_ which had 15.3% more precipitation. Finally, two drought indices represent different drought types. PDSI represents the meteorological drought and is based on an empirical method of a balance between moisture supply and demand^65^, while the MSDI is based on a probabilistic approach that is joint distribution of precipitation and soil moisture^54^.

### AquaCrop model evaluation

Supplementary Tables S4–S7 in the Supplementary information present the planting date, harvest index (HI), fertilizer stress (FS), calibration period, observed and simulated average yields, and RMSE for the AquaCrop simulations. The planting date of rainfed rice varies from April 20 to July 1, and it depends on the start of the wet season for each location^40^. HI is a function of water availability and crop variety^59^, and a higher value generates a higher rice yield. FS demonstrates deficit nutrient conditions in soil layers, and a higher value adversely affects the yield. For all provinces, HI varies from 23 to 37%, which was within the range of other applications in the LMB^40,59^. The mean values of RMSE for each country were 0.30, 0.30, 0.20, and 0.40 ton/ha for Cambodia, Lao PDR, Thailand, and Vietnam, respectively. The RMSE values were 15.6%, 9.7%, 8.4%, and 9.3% of the mean values of the simulated rice yields during the calibration period. For all provinces, RMSE varied from 0.10 to 0.61 ton/ha, which were 3.9–22.2% of the simulated rice yields, which were within the RMSE range of the previous studies in the LMB^40,67^. Therefore, the results implied that model performances of SWAT and AquaCrop were satisfactory for the simulation of rainfed rice in the LMB. Since those calibration parameters optimize the suitable planting date, water availability, and nutrient deficiency of the land, appropriate parameter estimations for each region are crucial for reliable simulations of the rice yields^40^. Furthermore, a well-calibrated hydrological model can be relied upon for accurate soil moisture and drought index computation, and the integrated hydrology model with crop simulation and climate models enables the assessment of future climate change impacts on drought and rice yields in the LMB.

### Relationship between soil moisture and rice yield

**Figure 4:**
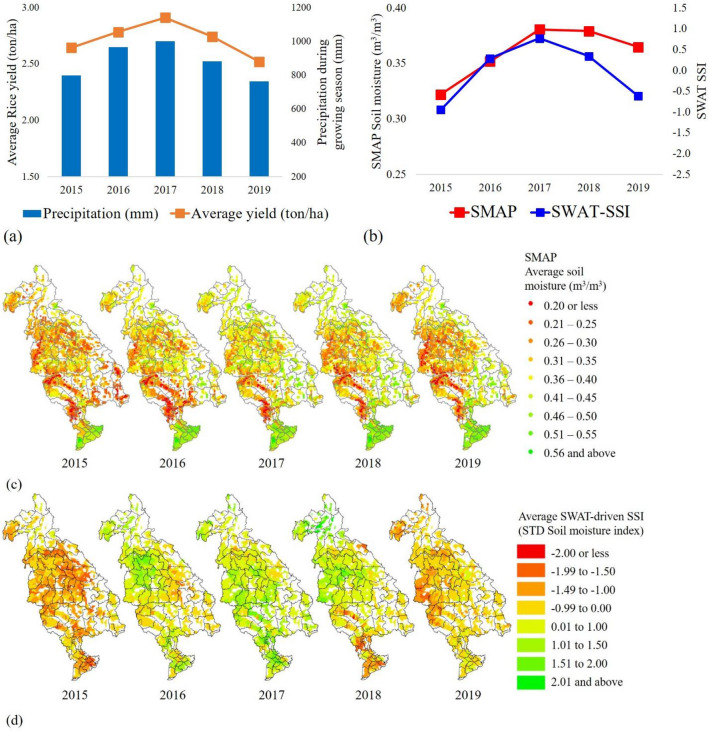
Comparisons of the SMAP soil moisture, model-driven drought index, and simulated rice yield for the last 5 years of the historical period (2015–2019). (**a**) Bar and line chart for precipitation and average rice yield. Blue bar represents the total precipitation amount during the crop growing season, and orange bar indicates the average rice yield for entire LMB. (**b**) Red line represents the average SMAP soil moisture during crop growing season, and blue line indicates the average model-driven SSI (Standardized Soil moisture Index: SSI) (**c**) Spatial maps of the average SMAP soil moisture (**d**) Spatial maps of the average model-driven SSI. Spatial maps were created using ArcMAP10.5 software by Esri (www.esri.com).

Figure 4a,b present the average SMAP soil moisture, average standardized soil moisture index (SSI), rice yield, and precipitation amount during the crop growing season. SMAP soil moisture and model-driven SSI showed similar patterns for drier years such as 2015 and 2019, while a slight overestimation of SWAT-SSI for an average rainfall year is seen. General ascent in wetness was seen in SMAP soil moisture, SSI, precipitation, and that corresponded well with increases in rice yield from 2015 to 2017, and decreases from 2017 to 2019. Figure 4c,d show spatial maps of the average soil moisture from SMAP and SSI for each year. Despite localized anomalies, the larger Mekong region including the delta did not suffer significant soil moisture deficits due to low climate variability and better surface water management projects, which led to stable rice yields (Supplementary Fig. S4d in the Supplementary Information). However, some regions showed fluctuation in rice yield and soil moisture patterns primarily driven by year-to-year monsoonal variability^68^. Those results imply that rainfed rice productions were primarily affected by precipitation and soil moisture conditions.

### Climate change impacts on droughts and rice yields

**Figure 5:**
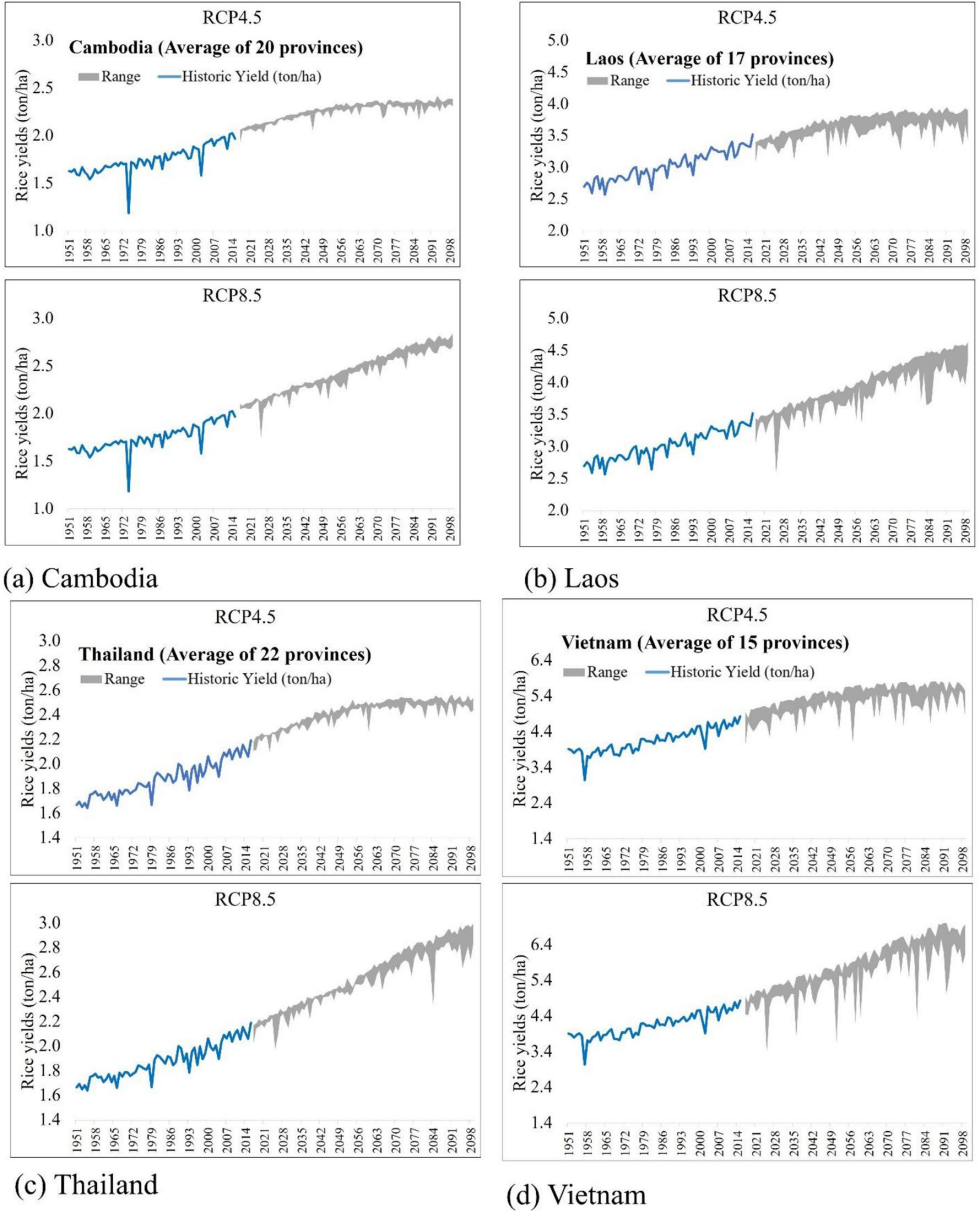
Time series of simulated rice yields for (**a**) Cambodia, (**b**) Lao PDR, (**c**) Thailand, and (**d**) Vietnam. X-axis represents the historical and future periods (1951–2099), and Y-axis indicates the rice yields (ton/ha). Blue lines present the average simulated rice yields for the historical period, and gray areas present the range of the simulated rice yields from the four climate models.

To further explore the future scenarios of production of rice, this study simulates long-term rice yield for the rainfed areas in the LMB, and Fig. 5 presents the time series of annual average simulated rice yield for the basin countries during the historical and future periods. During the historical period, trends in rice yields had a gradual increase. While comparing the earliest decade (1956–1965) and the recent decade (2006–2019), there were increases in rice yield up to 0.34, 0.52, 0.35, and 0.84 ton/ha for Cambodia, Lao PDR, Thailand, Vietnam, respectively. However, there were some sharp declines in rice yield. Supplementary Figure S5 in the Supplementary information shows the yearly rice yields and the total amount of precipitation during the crop growing season, and black and dashed circles highlight the drops in rice yield triggered by the reduced amount of precipitation. For instance, Supplementary Fig. S5a shows some sudden decline in rice yield due to decreases in precipitation in Cambodia, highlighting the fragile dependency of rainfed agriculture in this region. For the historical period, average total precipitation during the crop growing season in Cambodia was 822 mm, but the precipitation totals in 1974 and 2002 were 492 and 186 mm, respectively. In 1974 and 2002, rice yield reduced to 1.18 ton/ha and 1.58 ton/ha, which were lesser than the average rice yield during the historical period (1.76 ton/ha).

**Figure 6:**
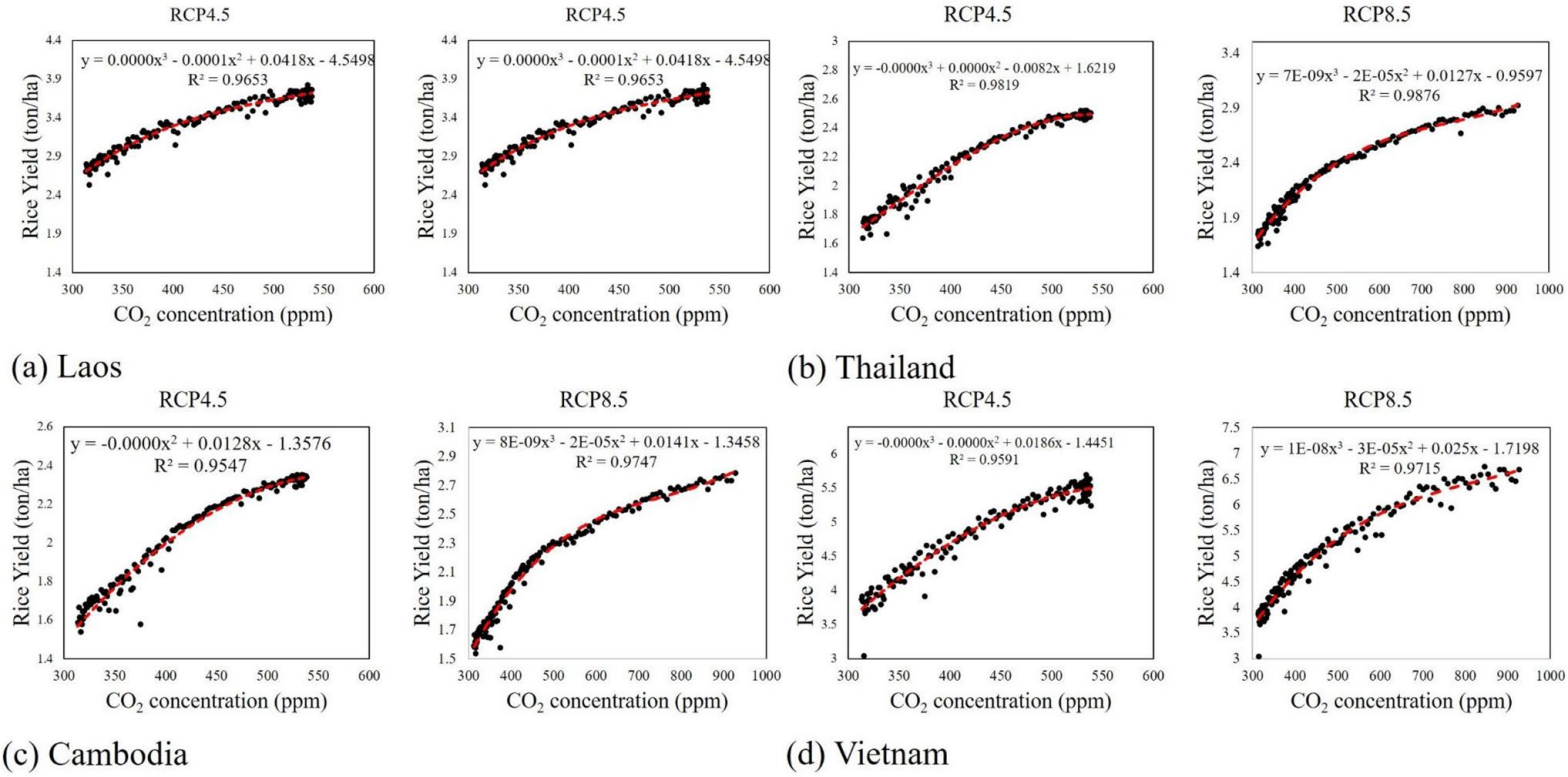
Non-linear plots of the CO_2_ concentration (X-axis) and rice yields (Y-axis) for (**a**) Lao PDR, (**b**) Thailand, (**c**) Cambodia, and (**d**) Vietnam. For each panel, the equations on the top show the polynomial relationships, and red and dashed lines are the fitted lines.

For the future periods, gradual increases in rice yield were projected, and the results of RCP8.5 showed a higher increase than that of RCP4.5. Compared to the recent decade of the historic period (2010–2019), there were increases in rice yields in the last decade of the twenty-first century (2090–2099). The increases were up to 20.2, 16.7, 18.1, and 21.4% under the RCP4.5 scenarios, and 41.8, 36.4, 39.4, and 45.4% under the RCP8.5 scenarios for Cambodia, Lao PDR, Thailand, and Vietnam, respectively. The fertilization effect of the CO_2_ increases potentially elevated the increases in rice yields under the RCP8.5 scenarios. The average CO_2_ concentration during the last decade of the historical period (2010–2019) was 399.4 ppm. Through the historical and future periods (1951–2099), CO_2_ concentration increased from 313.5 ppm to 538.4 ppm for RCP4.5 (71.7%) and 927.2 ppm for RCP8.5 (195.5%), and those increases derived 42.8% and 75.4% increases in rice yield on average for entire LMB. Besides, there was a non-linear relationship between rice yield and CO_2_ concentration. The average CO_2_ concentration for 2090–2099 were projected to be 536.3 ppm for RCP4.5 and 886.1 ppm for RCP8.5, and it amounted to about 34.3% and 121.9% increases compared to 2010–2019. These results are comparable with other studies that concluded increased crop yield in LMB^40,67^, by up to 25% due to increased CO_2_ concentration levels. Figure 6 presents non-linear scatter plots of crop yield and CO_2_ concentration, which show a strong positive relationship in all countries and for both RCP4.5 and RCP8.5. Numerous studies have pointed out a positive fertilization effect of the elevated CO_2_ concentration on crop production^69–71^. This study also confirms that CO_2_ concentration is a primary driver of biomass productivity and rice yield under climate change conditions. However, this study does not evaluate some detrimental impacts of elevated CO_2_ concentration on crop quality such as protein, micronutrients, and vitamin contents of rice^72,73^. Baker and Allen^74^ and Allen et al.^75^ also found the non-linear relationship between CO_2_ concentration and rice yield, and explained that elevated CO_2_ concentrations improved crop yields due to increased photosynthesis, and even further propounded that doubling of the CO_2_ concentration will increase crop yields up to 50%. Allen et al.^76^ tested a non-linear model to verify the impacts of the elevated CO_2_ concentration on crop yields and found proportional yield decreases under the high CO_2_ level that derived the non-linear relationship.

From the investigation of long-term projections of rice yield, it is clear that simulated rice yield was generally found to be increasing in all countries; however, there were some sharp declines in rice yields for the future periods. The numbers of drought events in the future periods were presented in Table 1, and the sharp declines in rice yield occurred during the drought years. Supplementary Figure S6 presents the spatial patterns of yield differences between the drought and non-drought years for future periods and climate models. As shown in the tables and figures, there was an overall reduction in rice yields during the drought years, and the decreases were highlighted as yellow to red areas. Specifically, in the VCHP (Vietnam Central High Plain) and some provinces Lao PDR regions, there were more than 0.6 ton/ha reductions in rice yields, which were derived from the sharp declines of precipitation during the crop growing seasons (Tables 1, 2). On the contrary, Cambodia and Mekong Delta showed less than 6.1% precipitation decreases during the drought year, and rice yields were relatively unchanged (− 0.15 to 0.3 ton/ha). With RCP4.5 projections, the mean values of the yield reductions for the f1 period were 0.041, 0.129, 0.057, 0.470, and 0.020 ton/ha, and for the f2 period were 0.039, 0.134, 0.044, 0.986, and 0.007 in Cambodia, Lao PDR, Thailand, VCHP, and Mekong Delta region, respectively. Similarly, from RCP8.5, the mean values of the yield reductions for the f1 period were 0.030, 0.152, 0.025, 0.960, and 0.014 ton/ha, and for the f2 period were 0.048, 0.128, 0.063, 0.484, and 0.018 in Cambodia, Lao PDR, Thailand, VCHP, and Mekong Delta region. Therefore, from the mean values of each country, this study found that lager yield reductions occurred in Laos PDR (− 0.128 to − 0.152 ton/ha) and VCHP (− 0.474 to − 0.986 ton/ha), while there were smaller scale reductions in the Mekong Delta (− 0.007 to − 0.020 ton/ha).

### Required irrigation areas and discussion

**Figure 7:**
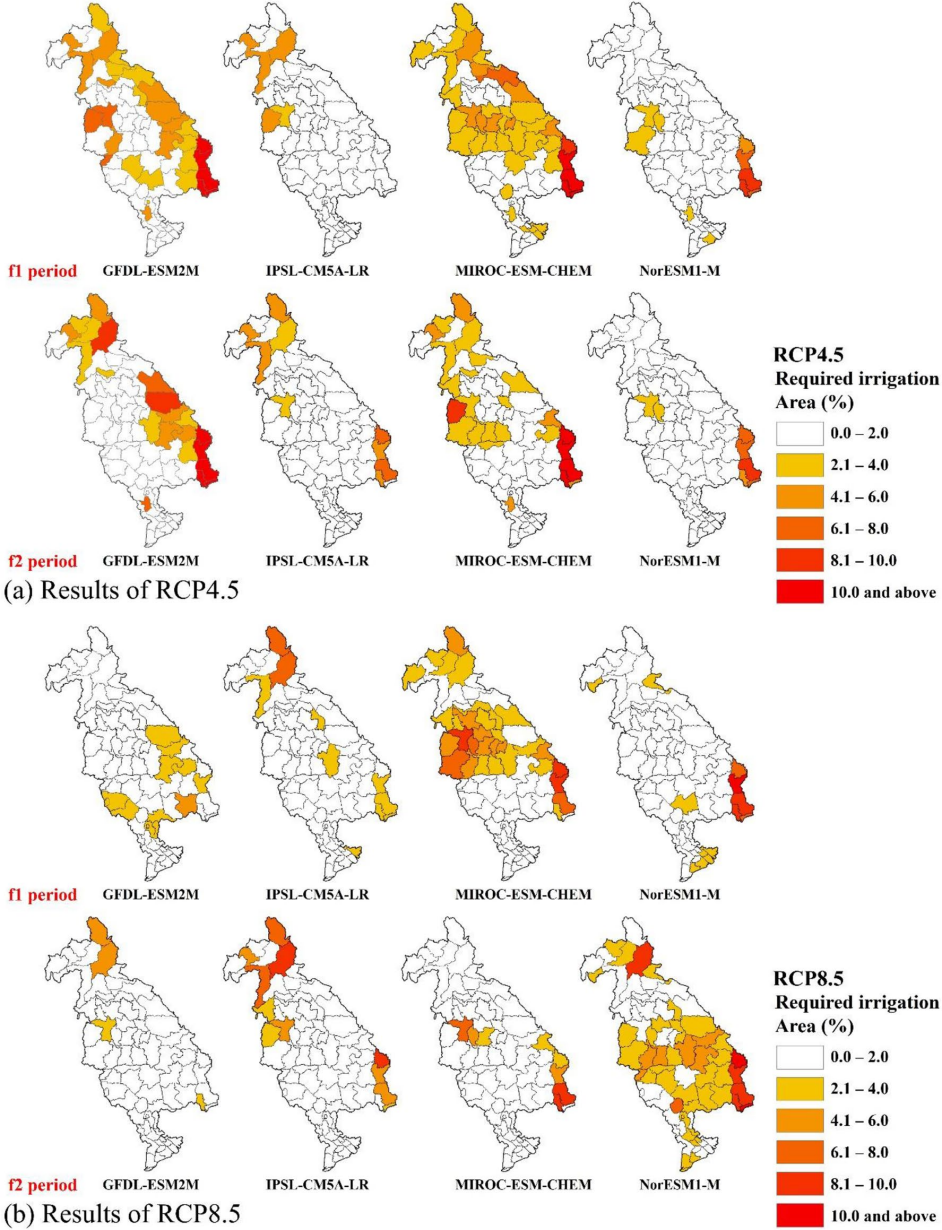
Required irrigation areas to compensate the yield decreases during drought years. Orange to red indicates the required areas. (**a**) Results of RCP4.5. (**b**) Results of RCP 8.5. Spatial maps were created using ArcMAP10.5 software by Esri (www.esri.com).

This study also explored an unconventional approach to mitigate the impacts of drought on food production by inverting yield loss to expanded areas for irrigation during drought years. Table 3 shows required irrigation areas (RIAs) computed both as area and percentage for paddy growing areas to compensate for the potential decreases in crop yields during the drought years, and Fig. 7 presents the spatial maps RIA. As defined in the “Methods” section, RIA is an additional irrigation area to compensate for the loss in rice yields during the drought years, and RIA% is the percentage of RIA needed to annex the existing paddy areas for each province (Eqs. 7, 8). and the highest mean values of RIA% was evident in the VCHP (up to 18.8%) and Lao PDR (up to 4.0%) regions, while the Mekong delta region showed the lowest (0.1–2.1%). The RIA% values were closely associated with the decreases in precipitation during the crop growing season, and the highest precipitation declines occurred in the VCHP region (− 15.3 to − 23.6%; Table 1). In addition, Lao PDR showed large decreases in precipitation (− 8.6 to − 13.6%). However, the Mekong delta showed lower RIA% as it might witness only moderate decreases (− 0.3%) or increases (0.2–2.0%) in precipitation.

**Table 3:** Required irrigation areas (RIA) and the percentage (RIA%) for the future periods. f1: 2020–2059, f2: 2060–2099.

RCPs	Countries	Rainfed paddy area (km^2^)	Average RIA and RIA%				GFDL-ESM2M		IPSL-CM5A-LR		MIROC-ESM-CHEM		NorESM1-M	
			f1		f2		f1	f2	f1	f2	f1	f2	f1	f2
			RIA (km^2^)	RIA%	RIA (km^2^)	RIA%	RIA%	RIA%	RIA%	RIA%	RIA%	RIA%	RIA%	RIA%
RCP 4.5	Cambodia	11,420	119	1.0	63	0.5	1.8	1.3	0.1	0.0	1.4	0.6	0.9	0.3
	Lao PDR	4590	105	2.3	94	2.0	3.7	4.0	1.5	1.2	3.3	2.3	0.7	0.7
	Thailand	32,220	516	1.6	300	0.9	2.0	1.0	0.6	0.5	2.8	1.7	1.1	0.5
	VCHP	643	63	9.8	68	10.6	18.8	17.8	0.6	5.2	12.5	12.7	7.3	6.9
	Mekong delta	9608	85	0.9	7	0.1	0.1	0.3	0.7	0.0	2.1	0.0	0.6	0.0
RCP 8.5	Cambodia	11,420	43	0.4	80	0.7	0.2	0.1	0.0	0.0	0.3	0.3	0.9	2.4
	Lao PDR	4590	49	1.1	67	1.5	0.1	0.8	1.6	2.1	2.1	0.6	0.4	2.4
	Thailand	32,220	400	1.2	343	1.1	0.0	0.2	0.6	0.7	4.2	1.0	0.2	2.3
	VCHP	643	28	4.3	33	5.1	0.3	0.5	2.6	4.2	6.0	5.0	8.3	10.6
	Mekong delta	9608	57	0.6	62	0.6	0.0	0.1	0.6	0.3	0.1	0.1	1.6	2.1

*RIA* Required irrigation area (km^2^), *RIA%* Required irrigation area (%), *VCHP* Vietnam Central High Plain.

From various climate projections of precipitation and temperature, which were used for soil moisture simulations by SWAT, the GFDL-ESM2M and MIROC-ESM-CHEM models presented drier and drought conditions resulting in higher RIA and RIA%, particularly the regions of Lao PDR, VCHP, western side of the LMB. In other words, higher values of RIA and RIA% are closely associated with reductions in precipitation during the crop growing season of the drought years. In the VCHP region, GFDL-ESM2M RCP4.5-based drought estimations projected the highest RIA% (17.8–18.8%; Fig. 7), which were deeply impacted by the reduced precipitation during the drought years (− 12.0 to − 15.4%; Table 3). Also, MIROC-ESM-CHEM-RCP4.5 resulted in high RIA% (12.5–12.7%), and they were also primarily due to decreases in precipitation during the drought years (− 29.8 to − 25.9%). On the contrary, MIROC-ESM-CHEM and NorESM1-M showed a smaller increase in RIA% (0.0–2.1%) that were caused by the modest precipitation declines (− 0.2 to − 3.0%) or increases (0.4–16.5%) in the Mekong Delta. Thus, the results imply that spatial heterogeneities and variabilities of precipitation between drought and non-drought years would lead to different responses in projected rice yields and areas in the LMB in the future, and higher uncertainties were probable in estimating the required irrigation areas as the climate model outputs were directly dependent on emission scenarios and climate models.

In the LMB, many dams have already been built and operated for various purposes (e.g., hydropower, irrigation, multi-purpose), and many dams are being planned in the future. In this study, the VCHP region was chosen as requiring the most RIA, and 28 dams were already commissioned for irrigation purposes. However, at least seven were also planned to be built for hydropower generation in the future^77^. Since the VCHP region needs the most RIA, an additional analysis was carried out to identify whether the additional dam for irrigation would fulfill the need. Besides, Lao PDR needs the second-largest RIA (up to 4.0% on average). However, since there were no currently operated or planned irrigation dams in the provinces, sound considerations and plans can potentially augment the need to meet crop production demand during the drought years.

Overall, the results of all climate models projected an increase in crop yields due to higher than historic precipitation and elevated CO_2_ concentration in the atmosphere. However, the climate models used in our study suggested probable decreases in precipitation during drought years that could result in drastic decreases in crop yields, especially in the VCHP and Lao PDR regions. For all climate models and future periods, an average of 55 and 39 provinces for RCP4.5 and RCP8.5 were affected by the decrease in precipitation during the crop growing season of the drought years. This also suggested that the future precipitation patterns estimated by the climate models could necessitate expansion in irrigated areas due to climate extremes, despite a long-term gradual increase in precipitation increases were possible. Understandably, these impacts can be mitigated by adjusting the planting date and applying adaptation methods^3^, while the uncertainties persisted in climate model predictions, future droughts, and rice production.

This study did not evaluate diverse adaptation options under various stresses including salinity intrusion, and dam constructions that were widely considered in other studies, and further analyses are required to assess their role in water and food security in the basin. Mainuddin et al.^40^ evaluated climate change impacts on rainfed rice yield in the LMB based on a crop growth simulation model (AquaCrop) and IPCC SRES A2 and B2 scenarios and reported the possibility for increased rice yield in Laos and Thailand due to the elevated CO_2_ concentration. They also assessed various adaptation methods such as supplementary irrigation, adjusting planting date, and reduction in fertility stress, which compensated negative impacts on rice yield by climate change. While it was not assessed in any earlier studies, our study evaluated the need for expanded irrigation areas for the entire LMB in the face of climate change-induced hydrologic extremes. Other studies also assessed adaptation strategies to mitigate the adverse impacts of climate change on rice production in Asia^39,78^, and found yield losses were primarily caused by heat stress and drought recommended that adjusting sowing dates would effectively compensate for the declines of rice yield. Also, further studies are required to estimate the impacts of additional irrigation on the water as well as energy budgets and managements^79–81^. Finally, sea-level rise and salinity intrusion in the Mekong Delta were not considered while significant sea-level rise (up to 30 cm in 2050) and salinity intrusion are expected, and extensive areas will be negatively affected^82,83^. Future dam constructions are also expected to cause reductions in downstream reaches flows in the basin^84^, and this could negatively impact rice production with a reduction in flows to the delta region^85^.

## Supplementary Information

Supplementary Information.

## Data Availability

Simulation data developed in this study will be made available on request to the corresponding author.

## References

[1] Fukai S, Sittisuang P, Chanphengsay M (1998). Increasing production of rainfed lowland rice in drought prone environments. Plant Prod. Sci..

[2] Parry MAJ, Flexas J, Medrano H (2005). Prospects for crop production under drought: Research priorities and future directions. Ann. Appl. Biol..

[3] Mainuddin M, Kirby M, Hoanh CT (2011). Adaptation to climate change for food security in the lower Mekong Basin. Food Secur..

[4] Dai A (2011). Drought under global warming: A review. Wiley Interdiscip. Rev. Clim. Change.

[5] Sheffield J, Wood EF (2012). Drought: Past Problems and Future Scenarios.

[6] Ashraf Vaghefi S, Mousavi SJ, Abbaspour KC, Srinivasan R, Yang H (2014). Analyses of the impact of climate change on water resources components, drought and wheat yield in semiarid regions: Karkheh River Basin in Iran. Hydrol. Process..

[7] Kang H, Sridhar V (2017). Combined statistical and spatially distributed hydrological model for evaluating future drought indices in Virginia. J. Hydrol. Reg. Stud..

[8] Kang H, Sridhar V (2018). Assessment of future drought conditions in the Chesapeake Bay watershed. JAWRA J. Am. Water Resour. Assoc..

[9] Cheeseman J, Khan MA, Ozturk M, Gul B, Ahmed MZ (2016). Food security in the face of salinity, drought, climate change, and population growth. Halophytes for Food Security in Dry Lands.

[10] USAID. *USAID Mekong ARCC Climate Change Impact and Adaptation Study for the Lower Mekong Basin (2013-2014) - Main Report*. Accessed 16 Apr 2021. https://www.usaid.gov/asia-regional/documents/usaid-mekong-climate-change-study-main-report-2013 (2013).

[11] Te, N. Drought management in the lower Mekong Basin. in *Southeast Asia Water Forum, 22-26 October 2007, Kuala Lumpur, Malaysia*. Accessed 16 Apr 2021. https://archive.iwlearn.net/mrcmekong.org/download/Papers/dmp-paper-seawfoct071.pdf (2007).

[12] Guo H (2017). Meteorological drought analysis in the Lower Mekong Basin using satellite-based long-term CHIRPS product. Sustain..

[13] Ruiz-Barradas A, Nigam S (2018). Hydroclimate variability and change over the Mekong River Basin: Modeling and predictability and policy implications. J. Hydrometeorol..

[14] MRC. *Annual Report 2019, Part 1, Progress and Achievements*. Accessed 16 Apr 2021. https://www.mrcmekong.org/assets/Publications/AR2019-Part-1.pdf (2019).

[15] Kang H, Sridhar V (2020). A near-term drought assessment using hydrological and climate forecasting in the Mekong River Basin. Int. J. Climatol..

[16] Nguyen NA (2017). Historic drought and salinity intrusion in the Mekong Delta in 2016: Lessons learned and response solutions. Viet. J. Sci. Technol. Eng..

[17] Office of the Resident Coordinator Viet Nam. Drought and saltwater intrusion. UN Viet Nam Situation Rep. 7. Accessed 16 Apr 2021. http://www.un.org.vn/en/publications/doc_details/526-viet-nam-drought-and-saltwater-intrusion-situation-report-no-7-as-of-25-october-2016.html (2016).

[18] Chen X, Liu H, Mu X (2020). Summary of flood and drought in Mekong River Basin. Flood Prevention and Drought Relief in Mekong River Basin.

[19] Adamson P, Bird J (2010). The Mekong: A drought-prone tropical environment?. Int. J. Water Resour. D..

[20] Thilakarathne M, Sridhar V (2017). Characterization of future drought conditions in the Lower Mekong River Basin. Weather Clim. Extremes.

[21] Cosslett, T. L. & Cosslett, P. D. The Lower Mekong Basin: Rice production, climate change, ENSO, and Mekong Dams. In *Sustainable Development of Rice and Water Resources in Mainland Southeast Asia and Mekong River Basin*. 85–114. 10.1007/978-981-10-5613-0 (Springer, Singapore, 2018).

[22] MRC. Assessment of basin-wide development scenarios: main report. Mekong River Commission, Vientiane (2011).

[23] FAOSTAT. Accessed 16 Apr 2021. http://www.fao.org/faostat/en/#data/ (2020).

[24] IRRI (International Rice Research Institute). World rice statistics online query facility. Accessed Apr 2020. http://ricestat.irri.org:8080/wrsv3/entrypoint.htm (2020).

[25] FAO. Food and Agriculture Organization of United Nations. Rice Market Monitor 2017 XX(4), 1–24. Accessed 16 Apr 2021. http://www.fao.org/3/i8317en/i8317en.pdf (2017).

[26] MRC. *Vulnerability Report Volume 2: Basin-wide Climate Change Impact and Vulnerability Assessment for Wetland Dependent Livelihoods and Eco-services*. (Mekong River Commission, Vientiane, 2015).

[27] Jalota SK (2012). Mitigating future climate change effects by shifting planting dates of crops in rice–wheat cropping system. Reg. Environ. Change.

[28] Kontgis C (2019). Climate change impacts on rice productivity in the Mekong River Delta. Appl. Geogr..

[29] Hoogenboom G (2010). Decision Support System for Agrotechnology Transfer (DSSAT) Version 4.5.

[30] Farquhar GD (1997). Carbon dioxide and vegetation. Science.

[31] Jiang Z (2019). Future changes in rice yields over the Mekong River Delta due to climate change—Alarming or alerting?. Theor. Appl. Climatol..

[32] Chun JA (2016). Assessing rice productivity and adaptation strategies for Southeast Asia under climate change through multi-scale crop modeling. Agric. Syst..

[33] Poulton PL, Dalgliesh NP, Vang S, Roth CH (2016). Resilience of Cambodian lowland rice farming systems to future climate uncertainty. Field Crops Res..

[34] Boonwichai S, Shrestha S, Babel MS, Weesakul S, Datta A (2018). Climate change impacts on irrigation water requirement, crop water productivity and rice yield in the Songkhram River Basin, Thailand. J. Clean. Prod..

[35] Trisurat Y, Aekakkararungroj A, Ma HO, Johnston JM (2018). Basin-wide impacts of climate change on ecosystem services in the Lower Mekong Basin. Ecol. Res..

[36] Bastakoti RC, Gupta J, Babel MS, van Dijk MP (2014). Climate risks and adaptation strategies in the Lower Mekong River basin. Reg. Environ. Change.

[37] Arnold JG (2012). SWAT: Model use, calibration, and validation. Trans. ASABE.

[38] Raes D, Steduto P, Hsiao T, Fereres E (2018). AquaCrop Version 6.0–6.1: Reference Manual.

[39] Li T, Angeles O, Radanielson A, Marcaida M, Manalo E (2015). Drought stress impacts of climate change on rainfed rice in South Asia. Clim. Change.

[40] Mainuddin M, Kirby M, Hoanh CT (2013). Impact of climate change on rainfed rice and options for adaptation in the lower Mekong Basin. Nat. Hazards.

[41] Jain VK, Pandey RP, Jain MK (2015). Spatio-temporal assessment of vulnerability to drought. Nat. Hazards.

[42] Kang H, Sridhar V (2018). Improved drought prediction using near real-time climate forecasts and simulated hydrologic conditions. Sustain..

[43] Sehgal V, Sridhar V (2019). Watershed-scale retrospective drought analysis and seasonal forecasting using multi-layer, high-resolution simulated soil moisture for Southeastern US. Weather Clim. Extremes.

[44] Danielson JJ, Gesch DB (2011). Global Multi-Resolution Terrain Elevation Data 2010 (GMTED2010).

[45] FAO (1995). Digital Soil Map of the World and Derived Soil Properties.

[46] USGS. USGS EROS Archive—Land Cover Products—Global Land Cover Characterization (GLCC). https://www.usgs.gov/centers/eros/science/usgs-eros-archive-land-cover-products-global-land-cover-characterization-glcc?qt-science_center_objects=0#qt-science_center_objects. Accessed 2020. (2020).

[47] Yatagai A (2012). APHRODITE: Constructing a long-term daily gridded precipitation dataset for Asia based on a dense network of rain gauges. Bull. Am. Meteorol. Soc..

[48] Sheffield J, Goteti G, Wood EF (2006). Development of a 50-year high-resolution global dataset of meteorological forcings for land surface modeling. J. Clim..

[49] Chen M (2008). Assessing objective techniques for gauge-based analyses of global daily precipitation. J. Geophys. Res. Atmos..

[50] Sridhar V, Kang H, Ali SA (2019). Human-induced alterations to land use and climate and their responses for hydrology and water management in the Mekong River Basin. Water.

[51] Pirmoradian N, Davatgar N (2019). Simulating the effects of climatic fluctuations on rice irrigation water requirement using AquaCrop. Agric. Water Manag..

[52] Mainuddin M, Kirby M (2009). Spatial and temporal trends of water productivity in the lower Mekong River Basin. Agric. Water Manag..

[53] Nazarenko L (2015). Future climate change under RCP emission scenarios with GISS ModelE2. J. Adv. Model. Earth Syst..

[54] Hao Z, AghaKouchak A (2014). A nonparametric multivariate multi-index drought monitoring framework. J. Hydrometeorol..

[55] Gringorten II (1963). A plotting rule for extreme probability paper. J. Geophys. Res..

[56] Abbaspour KC (2011). User Manual for SWAT-CUP: SWAT Calibration and Uncertainty Analysis Programs.

[57] Nash JE, Sutcliffe JV (1970). River flow forecasting through conceptual models part I—A discussion of principles. J. Hydrol..

[58] Yao F, Xu Y, Lin E, Yokozawa M, Zhang J (2007). Assessing the impacts of climate change on rice yields in the main rice areas of China. Clim. Change.

[59] Hayashi S, Kamoshita A, Yamagishi J, Kotchasatit A, Jongdee B (2007). Genotypic differences in grain yield of transplanted and direct-seeded rainfed lowland rice (*Oryza sativa* L.) in northeastern Thailand. Field Crops Res..

[60] Sridhar V (2013). Evaluating bias-corrected AMSR-E soil moisture using in situ observations and model estimates. Vadose Zone J..

[61] Bradford JB (2017). Future soil moisture and temperature extremes imply expanding suitability for rainfed agriculture in temperate drylands. Sci. Rep..

[62] Reichle, R. *et al. SMAP L4 Global 3-hourly 9 km EASE-Grid Surface and Root Zone Soil Moisture Geophysical Data, Version 5. [Indicate subset used]*. NASA National Snow and Ice Data Center Distributed Active Archive Center. 10.5067/9LNYIYOBNBR5 (2020).

[63] Hempel S, Frieler K, Warszawski L, Schewe J, Piontek F (2013). A trend-preserving bias correction—The ISI-MIP approach. Earth Syst. Dyn..

[64] Moriasi DN (2007). Model evaluation guidelines for systematic quantification of accuracy in watershed simulations. Trans. ASABE.

[65] Dai A, Trenberth KE, Qian T (2004). A global dataset of Palmer Drought Severity Index for 1870–2002: Relationship with soil moisture and effects of surface warming. J. Hydrometeorol..

[66] Chen M, Xie P, Janowiak JE, Arkin PA (2002). Global land precipitation: A 50-yr monthly analysis based on gauge observations. J. Hydrometeorol..

[67] Mainuddin M, Kirby M, Hoanh CT (2012). Water productivity responses and adaptation to climate change in the lower Mekong basin. Water Int..

[68] Zhang B (2014). Drought impact on vegetation productivity in the Lower Mekong Basin. Int. J. Rem. Sens..

[69] Krishnan P, Swain DK, Bhaskar BC, Nayak SK, Dash RN (2007). Impact of elevated CO_2_ and temperature on rice yield and methods of adaptation as evaluated by crop simulation studies. Agric. Ecosyst. Environ..

[70] Vanuytrecht E, Raes D (2011). Assessment of the ‘CO_2_ fertilization effect’ on crops with the AquaCrop model. Geophys. Res. Abstr..

[71] Deryng D (2016). Regional disparities in the beneficial effects of rising CO_2_ concentrations on crop water productivity. Nat. Clim. Change.

[72] Erda L (2005). Climate change impacts on crop yield and quality with CO_2_ fertilization in China. Philos. Trans. R. Soc. B. Biol. Sci..

[73] Zhu C (2018). Carbon dioxide (CO_2_) levels this century will alter the protein, micronutrients, and vitamin content of rice grains with potential health consequences for the poorest rice-dependent countries. Sci. Adv..

[74] Baker JT, Allen LH (1993). Effects of CO_2_ and temperature on rice. J. Agric. Meteorol..

[75] Allen LH, Prasad PV, Goodman RM (2004). Crop responses to elevated carbon dioxide. Encyclopedia of Plant and Crop Science.

[76] Allen LH (1987). Response of vegetation to rising carbon dioxide: Photosynthesis, biomass, and seed yield of soybean. Glob. Biogeochem. Cycles.

[77] CGIAR. Greater Mekong CGIAR Research Program, Water Land Ecosystems (WLE) database. https://wle-mekong.cgiar.org/changes/our-research/greater-mekong-dams-observatory/. Accessed 14 Aug 2020 (2020).

[78] Ding Y, Wang W, Zhuang Q, Luo Y (2020). Adaptation of paddy rice in China to climate change: The effects of shifting sowing date on yield and irrigation water requirement. Agric. Water Manag..

[79] Jaksa WT, Sridhar V (2015). Effect of irrigation in simulating long-term evapotranspiration climatology in a human-dominated river basin system. Agric. For. Meteorol..

[80] Sridhar V, Anderson KA (2017). Human-induced modifications to land surface fluxes and their implications on water management under past and future climate change conditions. Agric. For. Meteorol..

[81] Sridhar V (2013). Tracking the influence of irrigation on land surface fluxes and boundary layer climatology. J. Contemp. Water Res. Educ..

[82] Smajgl A (2015). Responding to rising sea levels in the Mekong Delta. Nat. Clim. Change.

[83] Vu DT, Yamada T, Ishidaira H (2018). Assessing the impact of sea level rise due to climate change on seawater intrusion in Mekong Delta, Vietnam. Water Sci. Technol..

[84] ICEM (International Center for Environmental Management). MRC Strategic Environmental Assessment (SEA) of hydropower on the Mekong mainstream—Final report, Hanoi, Viet Nam (2010).

[85] Cosslett TL, Cosslett PD (2014). Major threats to Mekong Delta: Climate change and mainstream dams. Water Resources and Food Security in the Vietnam Mekong Delta.

