# Supplementary material for: Future rice farming threatened by drought in the Lower Mekong Basin
Source: Sci Rep. 2021 Apr 30;11:9383. doi: 10.1038/s41598-021-88405-2 (PMC8087702; doi:10.1038/s41598-021-88405-2)
Supplement: Supplementary file 1 — Supplementary Information. [file 41598_2021_88405_MOESM1_ESM.docx]

Supplement information

**Future rice farming threatened by drought in the Lower Mekong Basin**

Hyunwoo Kang^1^, Venkataramana Sridhar^1*^, Mohammed Mainuddin^2^, Le Duc Trung^3^

^1^ Department of Biological Systems Engineering, Virginia Tech, Blacksburg, VA 24061, U.S.A

^2^ Water Security Program | CSIRO Land and Water, Black Mountain Laboratories, GPO Box 1700, Canberra ACT 2601, Australia

^3^ Viet Nam National Mekong Committee, 23 Hang Tre, Ha Noi, Viet Nam

*(Correspondence to vsri@vt.edu)

The SWAT model was calibrated by adjusting model parameters, and Table S1 presents them. For the SWAT model, Sridhar et al. (2019) developed calibration parameters using the SWAT calibration and uncertainty assessment tool (SWAT-CUP; Abbaspour, 2011), and they were used in this study. The calibration parameters include the basic hydrologic properties of the basin.

Table S1. Description of calibration parameters for the soil and water assessment tool (SWAT)

| Model | Parameter | Description | Min | Max |
| --- | --- | --- | --- | --- |
| SWAT | r_CN2.mgt | Curve number for moisture condition II | -0.2 | 0.2 |
|  | v_ALPHA_BF.gw | Baseflow alpha factor | 0 | 1 |
|  | v_GW_DELAY.gw | Groundwater delay time | 30 | 450 |
|  | v_GWQMN.gw | Threshold water depth in shallow aquifer for back discharge | 0 | 2000 |

Model-driven drought conditions (MSDI) were validated with the comparisons of the reference drought index at the moderate to extreme drought category (PDSI<-2, MSDI<-1), and Table S2 presents the drought categories of PDSI and MSDI. Overall, a smaller negative value indicates the severe drought conditions, while a larger positive value represents the wetter conditions.

Table S2. Classification of drought categories (Palmer, 1965, Hao and AghaKouchak, 2014)

| MSDI | PDSI | Drought categories |
| --- | --- | --- |
| -2.0 or below | -4.0 or below | Extreme drought |
| -1.50 to -1.99 | -3.0 to -3.99 | Severe drought |
| -1.0 to -1.49 | -2.0 to -2.99 | Moderate drought |
| -0.99 to 0.99 | -1.99 to 1.99 | Near normal |
| 1.0 to 1.49 | 2.0 to 2.99 | Moderately wet |
| 1.5 to 1.99 | 3.0 to 3.99 | Very wet |
| 2.0 and above | 4.0 and above | Extremely wet |

The SWAT model calibration was performed by comparing monthly streamflows at seven stations. Table S3 shows the results of the coefficient of determination (R^2^) and Nash and Sutcliffe (NS) efficiency for the streamflow calibration, and all seven stations that showed values above 0.8, are considered ‘Very good’ for the monthly simulation (Moriasi et al., 2007).

Table S3. Results of streamflow calibration. NS: Nash and Sutcliffe efficiency, R^2^: Coefficient of determination

| Station | Calibration period | Validation period | Calibration | | Validation | |
| --- | --- | --- | --- | --- | --- | --- |
|  |  |  | R^2^ | NS | R^2^ | NS |
| 1. Chiang Saen | 1984-1990 | 1991-1996 | 0.92 | 0.86 | 0.93 | 0.85 |
| 2. Luang Prabang | 1984-1990 | 1991-1997 | 0.93 | 0.81 | 0.94 | 0.86 |
| 3. Vientiane | 1984-1990 | 1991-1996 | 0.92 | 0.83 | 0.95 | 0.88 |
| 4. Nakhon Phanom | 1984-1990 | 1991-1995 | 0.93 | 0.87 | 0.92 | 0.86 |
| 5. Mukdahan | 1984-1990 | 1991-1995 | 0.93 | 0.89 | 0.93 | 0.88 |
| 6. Pakse | 1984-1990 | 1991-1998 | 0.90 | 0.84 | 0.90 | 0.85 |
| 7. Kratie | 1984-1990 | 1991-1998 | 0.90 | 0.85 | 0.91 | 0.86 |

Long-term historical drought conditions (1954 - 2014) were validated by the comparisons of Palmer Drought Severity Index (PDSI) and Multivariate Standardized Drought Index (MSDI) at a moderate drought category (PDSI<-2, MSDI<-1). Figure S1 shows the selected assessment locations were highlighted as red points for PDSI and black areas for the model-driven drought index (MSDI). Based on the results of PDSI, the first location (Luang Namtha province, Lao PDR) experienced 49, 77, and 94 months of extreme, severe, and moderate droughts, and the most severe and extended drought occurred from 2009 to 2010. The second location (Xayaboury province, Lao PDR) experienced 13, 26, and 66 months of extreme, severe, and moderate droughts, and the most severe and long drought occurred from 1957 to 1959. The third location (Roi Et province, Thailand) experienced 6, 25, and 76 months of extreme, severe, and moderate droughts, and the most severe and extended drought occurred from 1957 to 1959. Lastly, there were 19, 37, and 70 months of extreme, severe, and moderate drought events in the fourth point (Siem Reap province, Cambodia), and the most severe and another extended drought occurred from 1977 to 1979. The mean values of four stations were 17.4, 33, and 61.2 months of extreme, severe, and moderate droughts based on the results of PDSI. Each category of the drought conditions was 2.4%, 4.5%, and 8.4% of the historical period. For each province, the most severe and extended drought occurred due to the anomalous precipitation decreases compared to the historical average, and they were -5.7%, -16.6%, -10.6%, and -5.1% for the Luang Namtha, Xayaboury, Roi Et, and Siem Reap provinces, respectively.

Figures S2 and S3 present the spatial illustration of the historical average and future changes of precipitation and temperature for each climate model. For the historical period, annual precipitation varied from 933 mm to 2,070 mm, and average temperature ranged from 21.3 °C to 28.8 °C. For the future periods, means of four climate models indicated that there were 23.2% to 29.3% increases in precipitation, and 0.29 °C to 5.71 °C increases in temperature for both RCPs and periods. In general, relative to the past, there were increases in precipitation with NorESM1-M and RCP8.5 - f2 producing the highest (47.0%) and the GFDL-ESM2M and RCP4.5 - f1 producing the lowest increase (18.3%). In addition, the highest temperature increases occurred in MIROC-ESM-CHEM and RCP8.5 - f2 (4.6 °C), while the lowest increase was NorESM1-M model and RCP8.5 - f2 (1.1 °C). Assessment of this broad range was necessary to bracket the uncertainty arising out the climate models. To understand the relationship between rice yield and precipitation, average annual yield and annual precipitation totals were plotted for the last five years of the historical period (2015 - 2019) (Figure S4). The Mekong delta region did not show a significant change in soil moisture due to a relatively constant precipitation amount that also led to stable rice yields. Since the period between 2015 and 2017 saw a general increase in precipitation, the slope of the yield line was ascending which became descending for 2018 and 2019. It is noteworthy that all the regions experienced a sharp decrease in crop yield in 2019 due to an extraordinary drought event.

Figure S5 presents the rice yields and the total amount of precipitation during the crop growing season for the historical period. Blue bars indicate the total amount of precipitation during the crop growing season (mm), orange lines indicate the simulated rice yield (ton/ha), and black and dashed circles highlight the sudden decreases of rice yields due to precipitation declines. For all four countries, rice yield was generally showing an increasing trend between 1956 and 2015. The average annual yields were 1.73, 2.98, 1.87, and 4.83 ton/ha for Cambodia, Lao PDR, Thailand, and Vietnam, respectively. A noteworthy feature of this analysis showed a dramatic drop in yield for the drought years, which highlighted the adverse impacts of year-to-year climate variability and the need for irrigation during those times to mitigate the crop loss. For example, in Cambodia, there were sudden drops in the rice yield in 1974 and 2002 due to the precipitation deficits. The rice yields were 1.18 and 0.58 ton/ha, which were much less than the average of the period (1.73 ton/ha).

Quantifying the differences in yield between the drought and non-drought years for future periods can be important to assess the range of scenarios as precipitation varied between climate models (Figure S6). Among the provinces, the highest yield reductions were projected from GFDL-ESM2M-RCP4.5 and MIROC-ESM-CHEM-RCP4.5 scenarios in Dak Lak in the VCHP region (-1.516 to -0.961 ton/ha). Although the average precipitation in the VCHP region increased for the GFDL-ESM2M-RCP4.5 and MIROC-ESM-CHEM-RCP4.5 models (18.2% to 25.9%; Figure S2), the significant reductions in precipitation during the crop growing season of the drought years (-34.4% to -12%) could be attributed to this reduction. On the other hand, relatively unchanged reductions in rice yields occurred in the Mekong delta provinces. For instance, the highest yield reduction was projected in the Can Tho province from NorESM1-M and RCP8.5 and f2 period (-0.234 ton/ha) due to the less reduction in precipitation during the drought year (-1.6%).


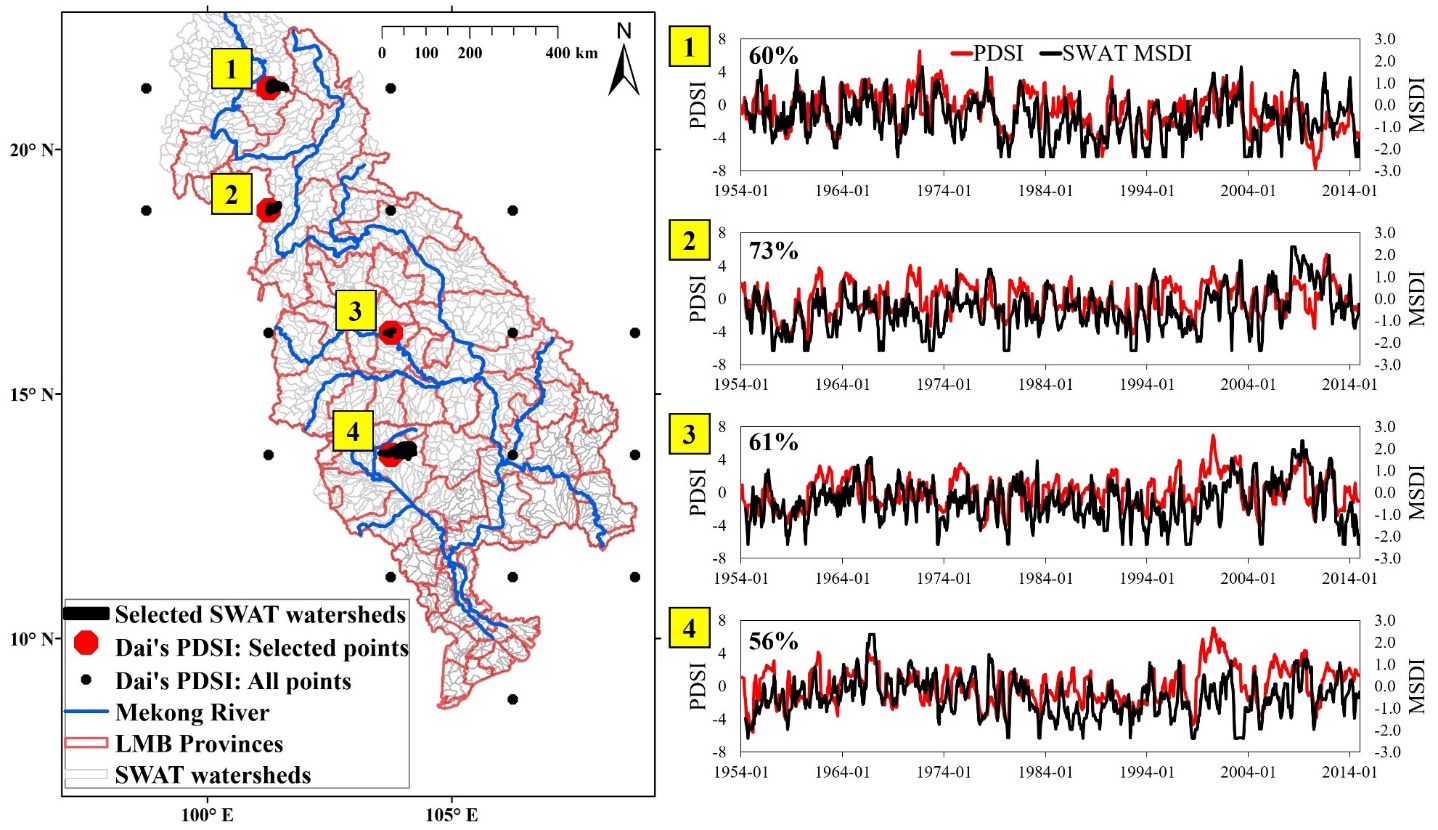


Figure S1. Comparisons of model-driven drought index (MSDI; Black lines) and the Palmer Drought Severity Index (PDSI; Red lines) for drought validations of the LMB. Spatial maps were created using ArcMAP10.5 software by Esri (www.esri.com).


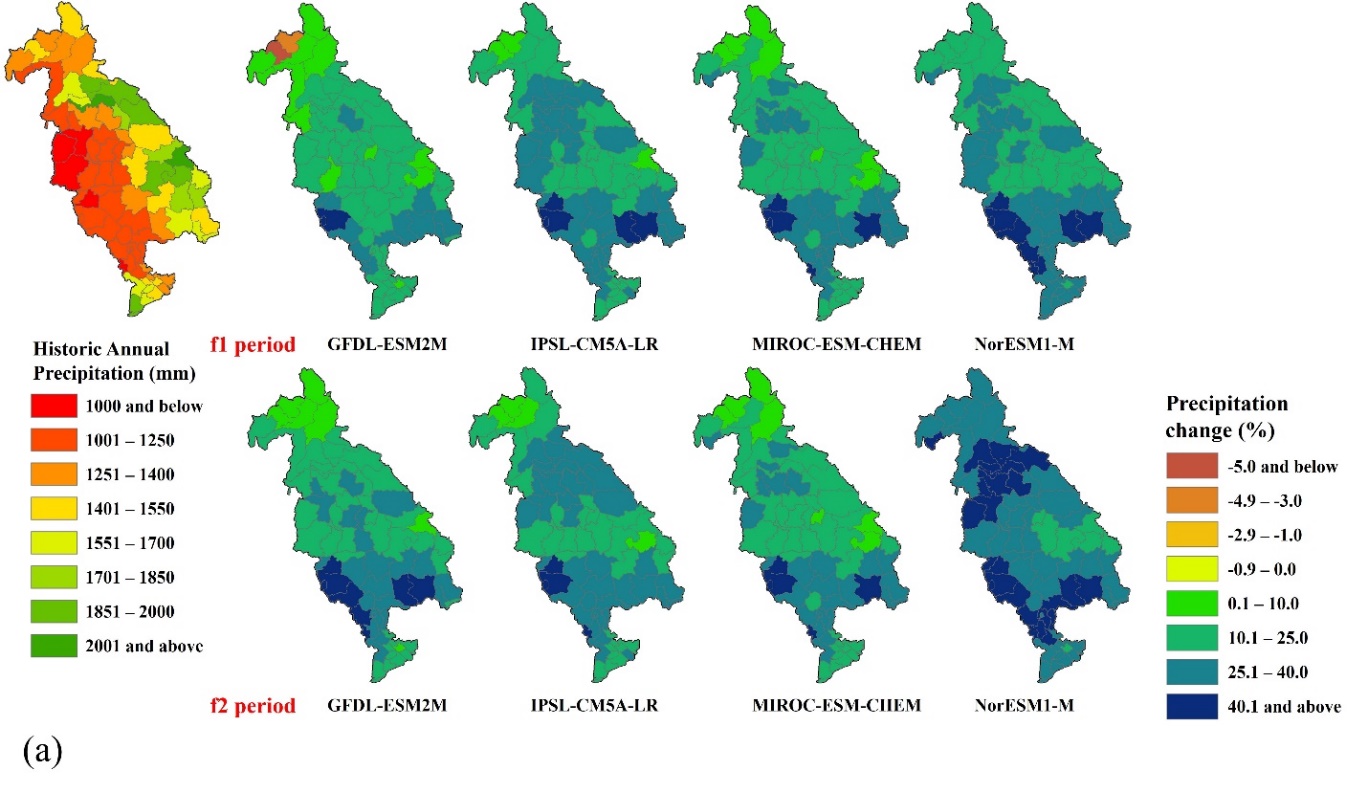


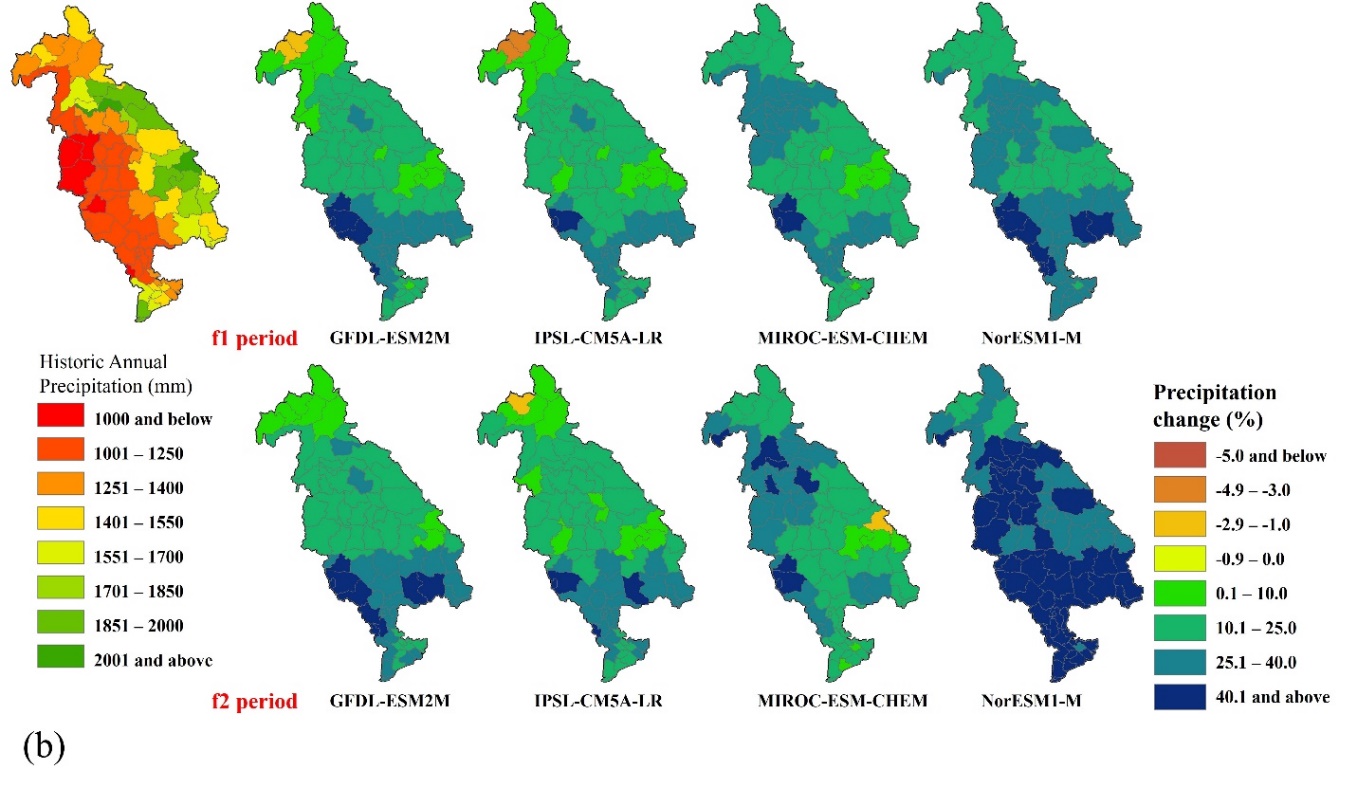


Figure S2. Spatial maps of the annual precipitation for the historical period (1954-2019) and changes in the future periods (%) (f1: 2020 – 2059, f2: 2060 – 2099). (a) Annual precipitation for the historical period (left figure) and precipitation changes for the RCP4.5 simulations. (b) Annual precipitation for the historical period (left figure) and precipitation changes for the RCP8.5 simulations. Spatial maps were created using ArcMAP10.5 software by Esri (www.esri.com).


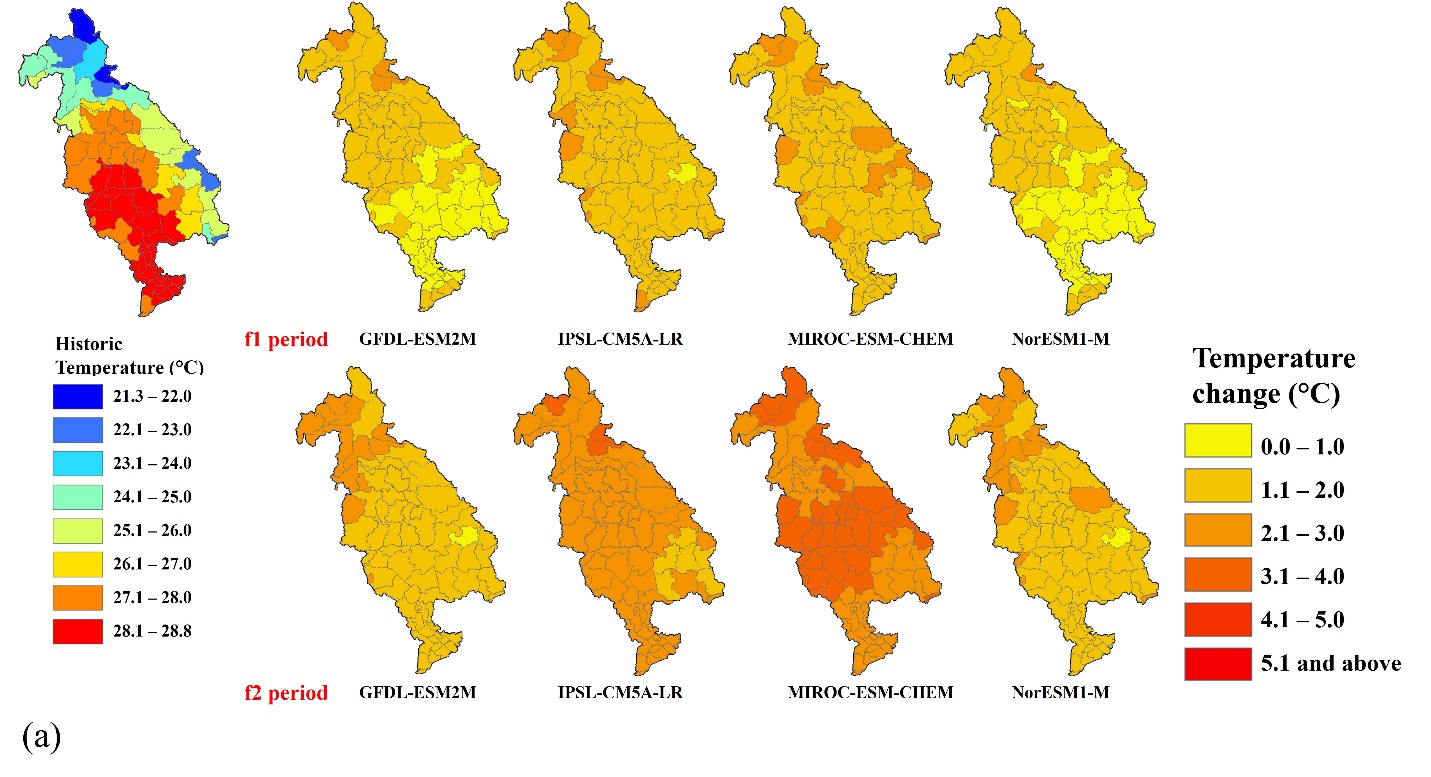


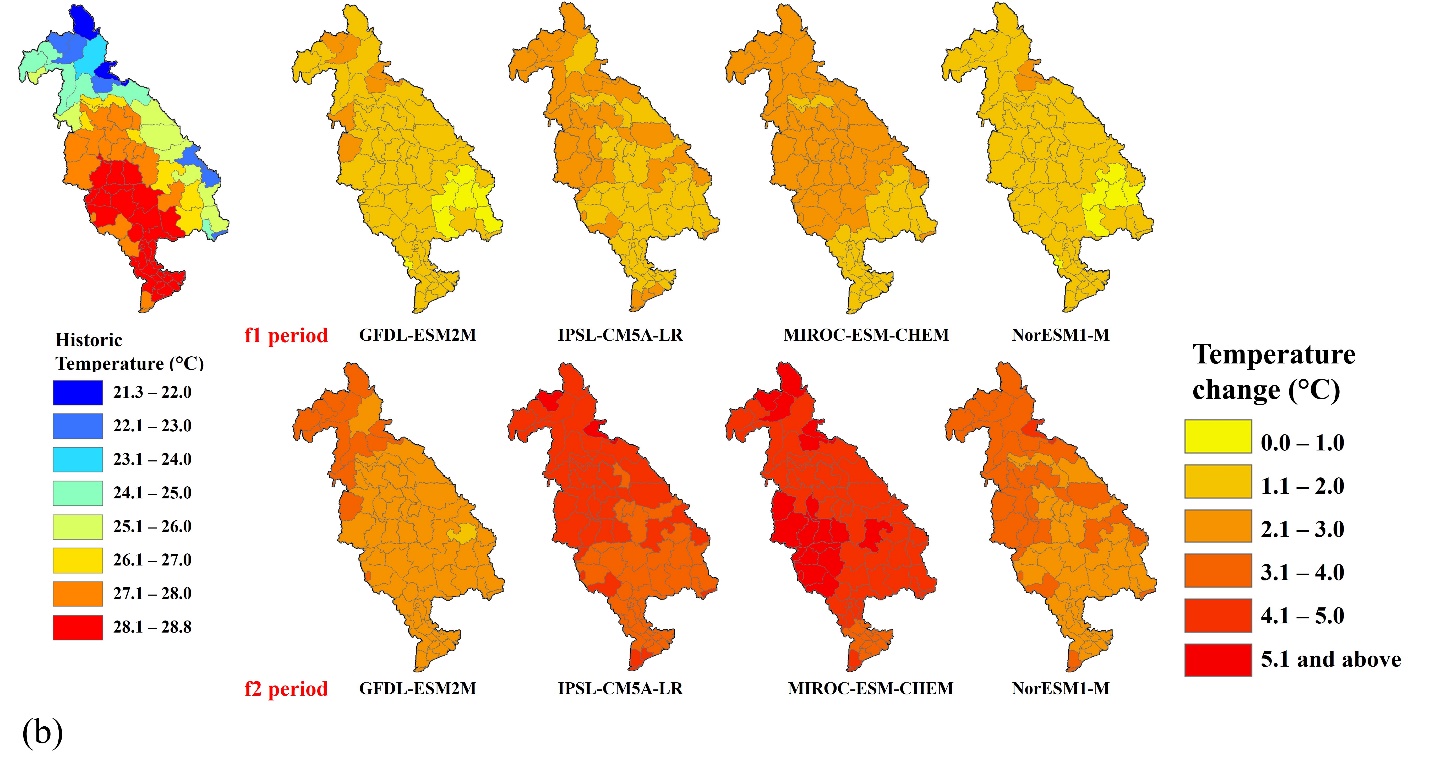


Figure S3. Spatial maps of the average temperature for the historical period (1954-2019) and changes in the future periods (°C) (f1: 2020 – 2059, f2: 2060 – 2099). (a) Average temperature for the historical period (left figure) and temperature changes for the RCP4.5 simulations. (b) Average temperature for the historical period (left figure) and temperature changes for the RCP8.5 simulations. Spatial maps were created using ArcMAP10.5 software by Esri (www.esri.com).


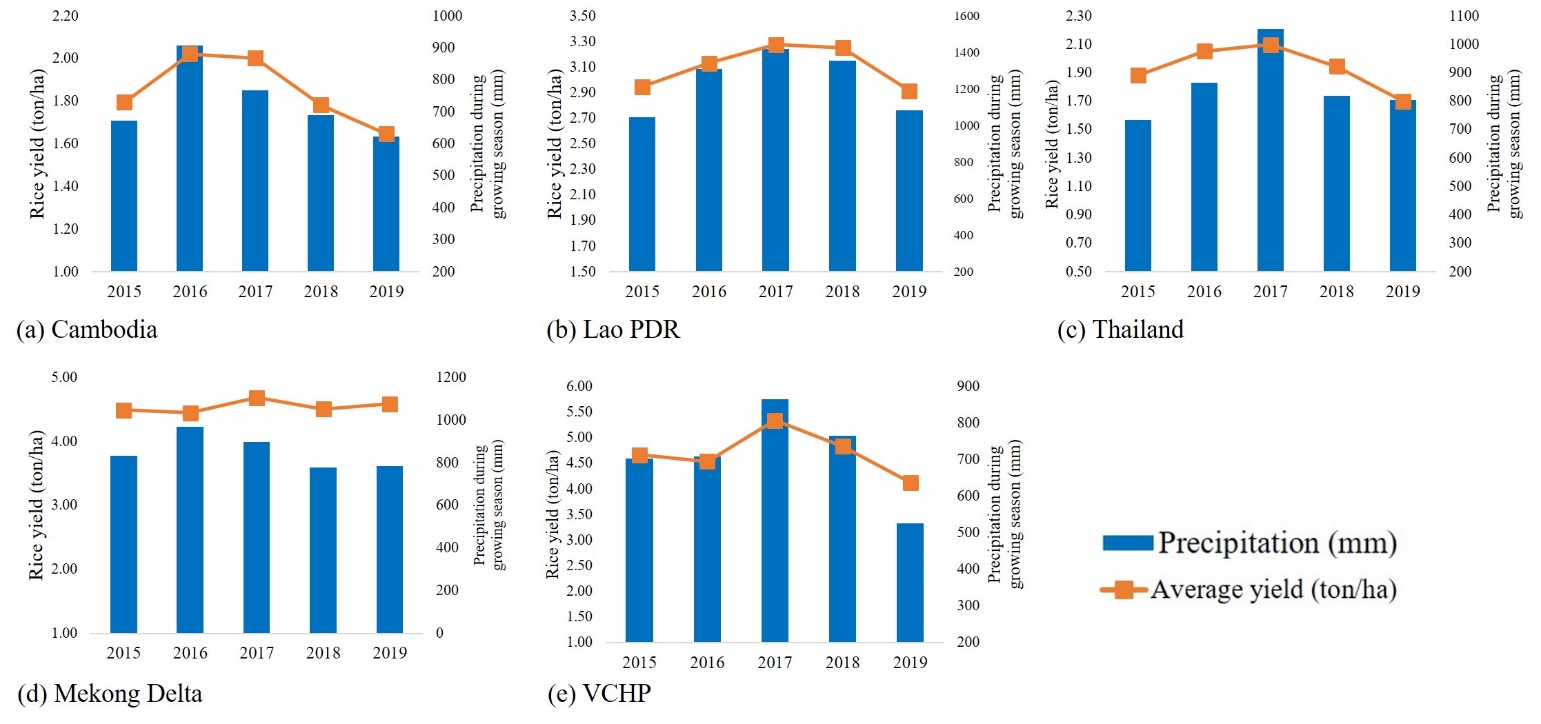


Figure S4. Bar and line chart for average rice yield and precipitation for the last five years of the historical period (2015 - 2019). Blue bar represents the total precipitation amount during the crop growing season, and orange bar indicates the average rice yield for entire LMB. (a) Cambodia (b) Lao PDR (c) Thailand (d) Mekong Delta (e) VCHP (Vietnam Central High Plain)


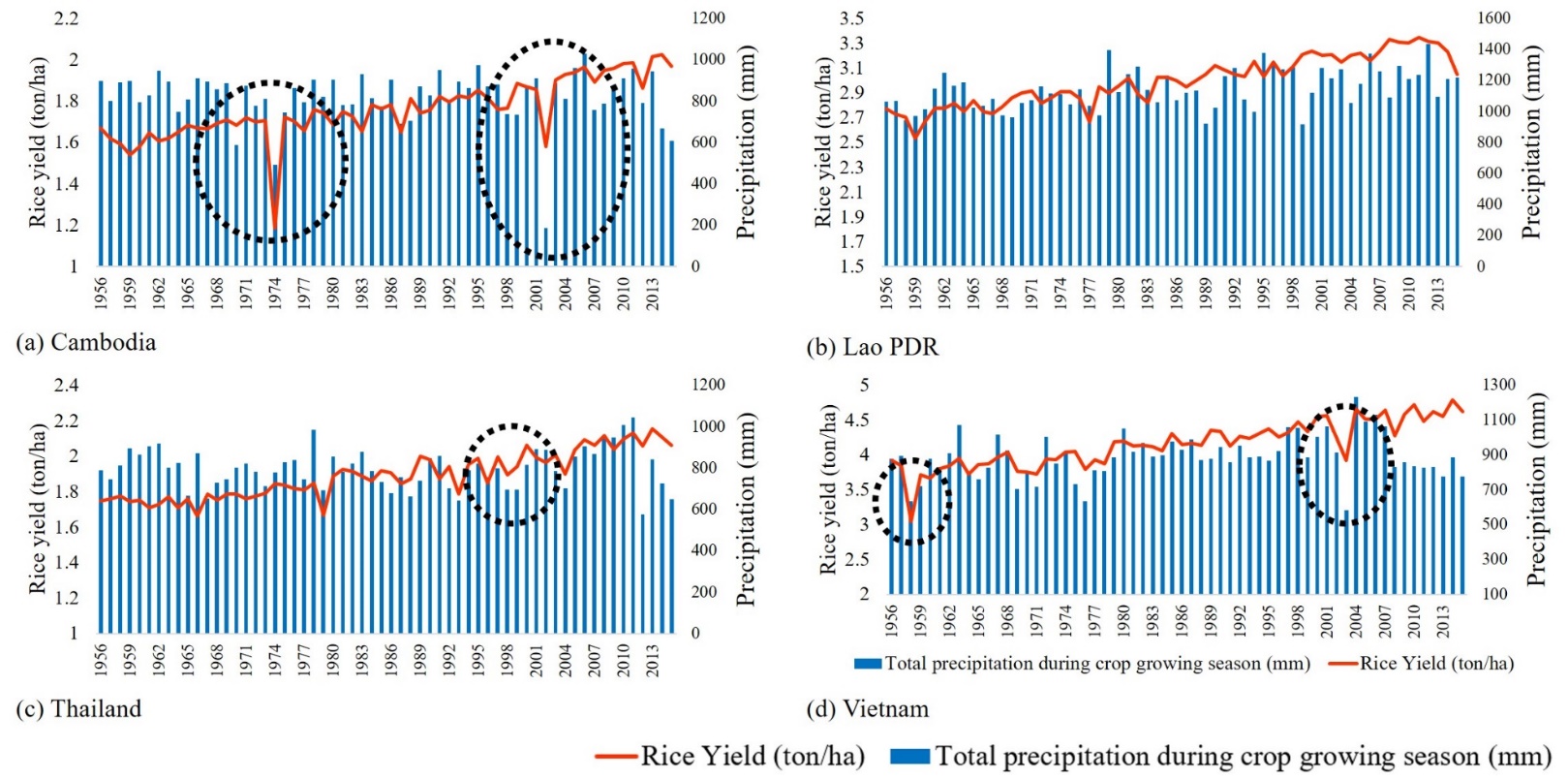


Figure S5. Time series of average rice yields for (a) Cambodia, (b) Lao PDR, (c) Thailand, and (d) Vietnam. Blue bars represent the total amount of precipitation during the crop growing season (mm), orange lines indicate the simulated rice yield (ton/ha), and black and dashed circles highlight the years of sudden decreases in precipitation and rice yield.


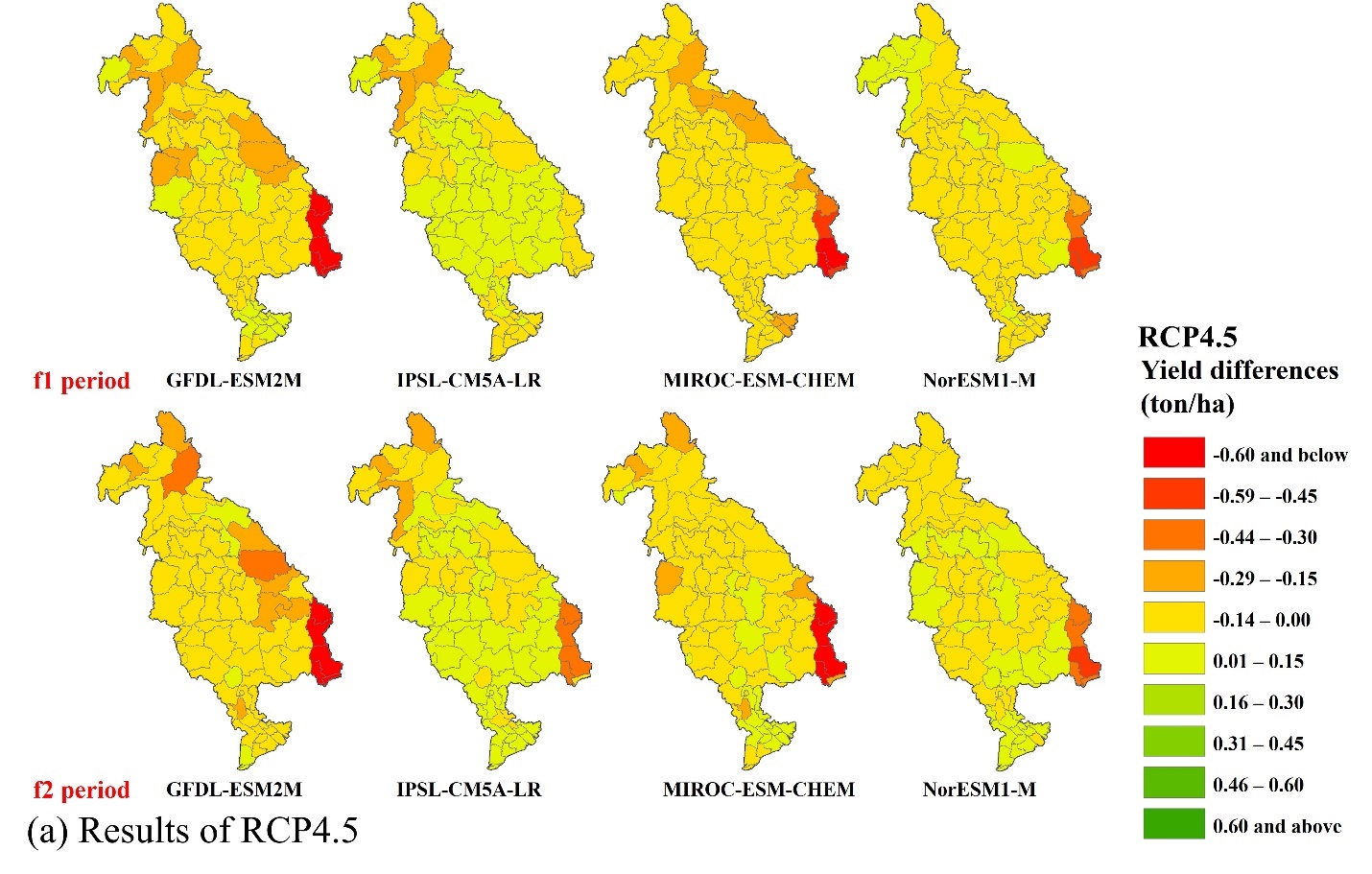


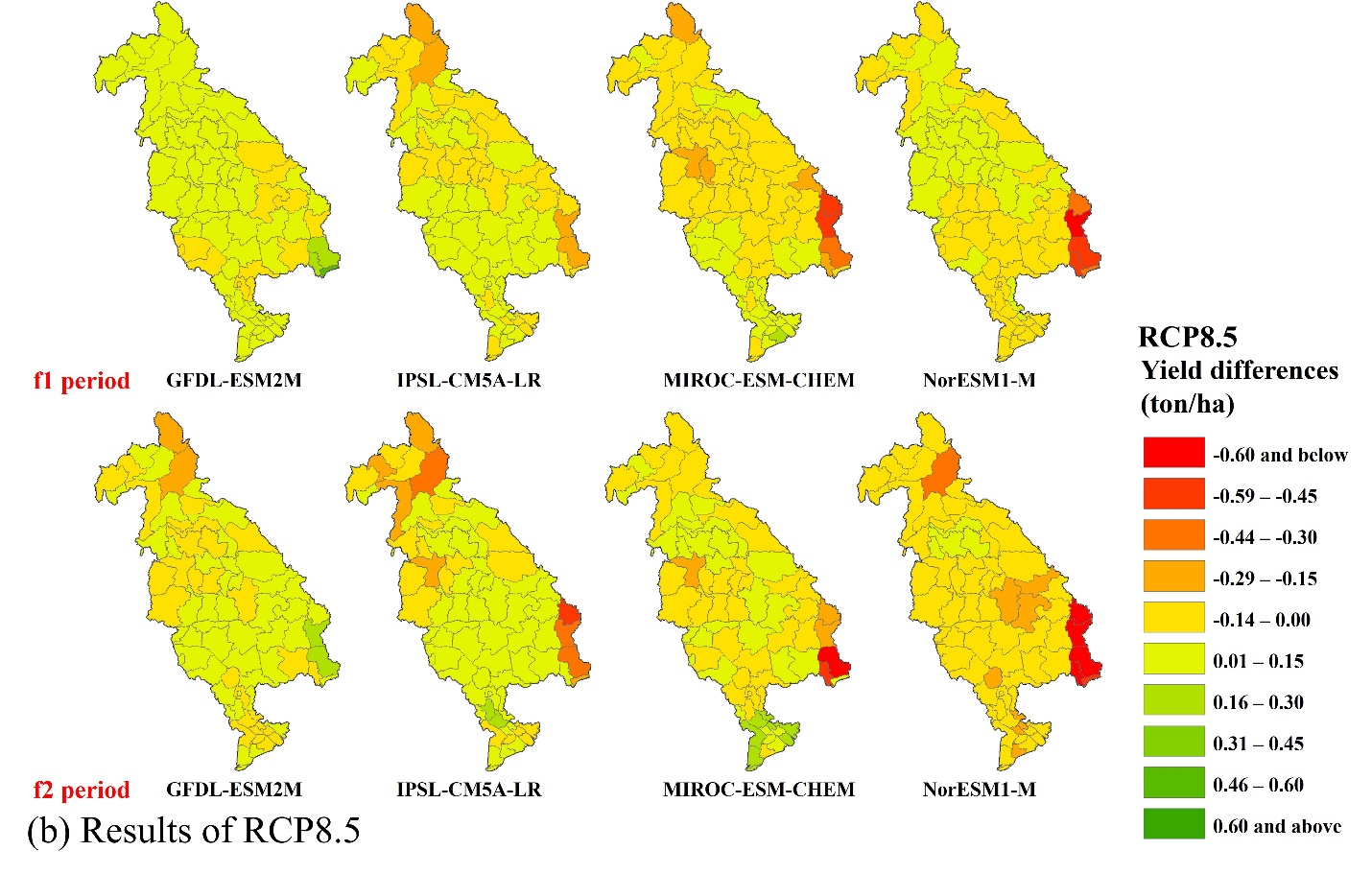


Figure S6. Differences of rice yield between the drought and non-drought years for each province. Light blue to green presents the increases in rice yields, but orange to red indicates the decreases in rice yields. (a) Results of RCP4.5. (b) Results of RCP8.5. Spatial maps were created using ArcMAP10.5 software by Esri (www.esri.com).

The model-driven rice yield from all provinces in the LMB were calibrated with observed rice yield. Planting date, harvest index (HI), fertilizer stress (FS) were used for the calibration parameters, and Root Mean Square Error (RMSE) was computed as a model evaluation criterion. Tables S4 to S7 show the planting date, HI, FS, calibration period, observed and simulated average yields, and RMSE for the AquaCrop simulations. The mean values of HI were 26.5%, 28.9%, 24.9%, and 32.2% for Cambodia, Lao PDR, Thailand, and Vietnam, respectively. In addition, the mean values of FS were 61.8%, 47.6%, 59.2%, and 27.8% for Cambodia, Lao PDR, Thailand, and Vietnam, respectively. The Gia Lai province in Vietnam was the highest average rice yield (5.27 ton/ha), and it was derived by the highest HI value (37%) and lower FS (22%). Besides, decreased precipitation during the crop growing season (869 mm) might have resulted in reductions in rice yield (4.71 ton/ha), while the higher precipitation (1,234 mm) led to a greater rice yield (5.71 ton/ha). The Siem Reap province in Cambodia showed the lowest average yield (1.51 ton/ha), and it was caused by the lowest HI value (24%) and higher FS (67%). Also, lesser precipitation during the crop growing season (817 mm) might have also caused decreased rice yield (1.47 ton/ha), while the higher precipitation (911 mm) could have produced a higher rice yield (1.55 ton/ha). The RMSE percentage in Cambodia was relatively higher (15.6%) because observed yields varied widely than in other countries. For instance, the difference in mean values between the highest and the lowest yield for Cambodia was 0.89 ton/ha, while it was 0.50 ton/ha in Thailand, where the average rice yield was similar to Cambodia but only had 8.4% of RMSE. The difference between the highest and lowest rice yield in Ubon Ratchathani province was the lowest among the 74 provinces (0.21 ton/ha), while An Giang province showed the 3^rd^ largest difference (2.01 ton/ha). Additionally, the difference was the highest in Dac Nong and Dak Lak provinces in Vietnam, and it was 2.34 ton/ha that was also linked with higher RMSE (0.60 ton/ha). The results imply that model is highly sensitive to the variability in observed rice yields. The range of RMSE was from 0.10 to 0.61 ton/ha, and they were 3.9% to 22.2% of the simulated rice yields for Ubon Ratchathani province in Thailand, and An Giang province in Vietnam, respectively. Since the range of RMSE was within the results from other studies in the LMB (Mainuddin et al., 2012; Mainuddin et al., 2013), the performance of the crop grow model was satisfactory for the rice simulation the LMB.

Table S4. Calibration parameters (planting date, harvest index, and fertilizer stress) used in the AquaCrop simulation and the average simulated rice yields for the Cambodia provinces. RMSE: Root mean square error (Calibration period: 1993 – 2001)

| Province | Planting date | Harvest index (%) | Fertilizer stress (%) | Observed average yield (t/ha) | Simulated average yield (t/ha) | RMSE (ton/ha) |
| --- | --- | --- | --- | --- | --- | --- |
| Kampong Spue | 20-Jun | 28 | 60 | 1.73 | 1.76 | 0.33 |
| Kampot | 10-Jun | 25 | 60 | 1.81 | 1.82 | 0.29 |
| Krong Pailin | 10-Jun | 26 | 62 | 1.90 | 1.90 | 0.33 |
| Pouthisat | 20-Jun | 27 | 63 | 1.83 | 1.89 | 0.33 |
| Batdambang | 20-Jun | 28 | 57 | 2.01 | 2.08 | 0.19 |
| Kampong Chhnang | 20-Jun | 26 | 59 | 1.78 | 1.82 | 0.30 |
| Rotano Kiri | 10-Jun | 24 | 63 | 1.52 | 1.52 | 0.32 |
| Stueng Traeng | 10-Jun | 24 | 61 | 1.58 | 1.60 | 0.16 |
| Takeo | 20-May | 30 | 53 | 2.03 | 2.01 | 0.40 |
| Kampong Cham | 20-Jun | 31 | 63 | 2.10 | 2.07 | 0.36 |
| Kampong Thom | 10-Jun | 25 | 66 | 1.62 | 1.64 | 0.26 |
| Kandal | 20-Jun | 30 | 50 | 2.84 | 2.85 | 0.37 |
| Kratie | 10-Jun | 28 | 66 | 1.88 | 1.90 | 0.25 |
| Mondul Kiri | 20-Jun | 24 | 69 | 1.51 | 1.53 | 0.34 |
| Phnom Penh | 10-Jun | 27 | 61 | 1.91 | 1.90 | 0.18 |
| Preah Vihear | 10-Jun | 24 | 68 | 1.53 | 1.54 | 0.21 |
| Prey Veng | 20-Jun | 28 | 65 | 1.86 | 1.86 | 0.30 |
| Banteay Meanchey | 20-Jun | 27 | 62 | 1.72 | 1.76 | 0.28 |
| Otdar Mean Chey | 20-Jun | 24 | 63 | 1.59 | 1.59 | 0.27 |
| Siemreab | 20-Jun | 24 | 67 | 1.48 | 1.51 | 0.25 |

Table S5. Calibration parameters (planting date, harvest index, and fertilizer stress) used in the AquaCrop simulation and the average simulated rice yields for the Lao PDR provinces. RMSE: Root mean square error (Calibration period: 1993 – 2004)

| Province | Planting date | Harvest index (%) | Fertilizer stress (%) | Observed average yield (ton/ha) | Simulated average yield (ton/ha) | RMSE (ton/ha) |
| --- | --- | --- | --- | --- | --- | --- |
| Attapeu | 20-May | 27 | 49 | 2.78 | 2.75 | 0.32 |
| Champasack | 20-May | 29 | 50 | 2.84 | 2.85 | 0.22 |
| Khammuane | 20-May | 28 | 50 | 2.79 | 2.75 | 0.33 |
| Saravan | 20-May | 30 | 47 | 3.02 | 3.07 | 0.34 |
| Savannakhet | 20-May | 30 | 46 | 3.17 | 3.18 | 0.19 |
| Sekong | 1-Jun | 28 | 48 | 3.06 | 3.08 | 0.29 |
| Bokeo | 1-Jun | 31 | 45 | 3.51 | 3.50 | 0.28 |
| Borikhamxay | 1-Jun | 30 | 53 | 2.84 | 2.88 | 0.49 |
| Luangprabang | 20-May | 31 | 49 | 3.29 | 3.26 | 0.25 |
| Phongsaly | 20-May | 27 | 46 | 3.08 | 3.08 | 0.45 |
| Vientiane | 1-Jun | 33 | 46 | 3.51 | 3.49 | 0.37 |
| Xaysomboon | 1-Jun | 27 | 49 | 2.98 | 3.00 | 0.20 |
| Luangnamtha | 15-Jun | 28 | 47 | 3.38 | 3.35 | 0.24 |
| Oudomxay | 15-Jun | 28 | 45 | 3.41 | 3.39 | 0.25 |
| VientianeC | 15-Jun | 29 | 45 | 3.36 | 3.34 | 0.33 |
| Xayabury | 1-Jun | 30 | 47 | 3.39 | 3.40 | 0.38 |
| Xiengkhuang | 15-Jun | 25 | 47 | 3.09 | 3.10 | 0.24 |

Table S6. Calibration parameters (planting date, harvest index, and fertilizer stress) used in the AquaCrop simulation and the average simulated rice yields for the Thailand provinces. RMSE: Root mean square error (Calibration period: 1995 – 2003)

| Province | Planting date | Harvest index (%) | Fertilizer stress (%) | Observed average yield (ton/ha) | Simulated average yield (ton/ha) | RMSE (ton/ha) |
| --- | --- | --- | --- | --- | --- | --- |
| Nakhon Phanom | 1-Jul | 25 | 60 | 1.77 | 1.80 | 0.21 |
| Ubon Ratchathani | 1-Jul | 24 | 61 | 1.64 | 1.66 | 0.10 |
| Amnat Charoen | 20-Jun | 24 | 62 | 1.77 | 1.78 | 0.14 |
| Mukdahan | 15-Jun | 24 | 59 | 1.87 | 1.87 | 0.25 |
| Nong Khai | 15-Jun | 24 | 61 | 1.86 | 1.89 | 0.16 |
| Sakon Nakhon | 15-Jun | 24 | 61 | 1.78 | 1.82 | 0.12 |
| Si Sa Ket | 15-Jun | 25 | 61 | 1.92 | 1.92 | 0.17 |
| Chiang Rai | 15-Jun | 28 | 49 | 2.88 | 2.92 | 0.15 |
| Kalasin | 20-Jun | 25 | 56 | 2.02 | 2.05 | 0.10 |
| Roi Et | 10-Jun | 24 | 59 | 1.85 | 1.88 | 0.13 |
| Surin | 10-Jun | 23 | 60 | 1.81 | 1.81 | 0.13 |
| Udon Thani | 10-Jun | 24 | 61 | 1.81 | 1.84 | 0.13 |
| Yasothon | 20-Jun | 24 | 62 | 1.71 | 1.74 | 0.15 |
| Buri Ram | 10-Jun | 24 | 59 | 1.87 | 1.89 | 0.11 |
| Chaiyaphum | 10-Jun | 24 | 60 | 1.84 | 1.85 | 0.22 |
| Khon Kaen | 1-Jun | 25 | 61 | 1.84 | 1.79 | 0.18 |
| Loei | 10-Jun | 28 | 56 | 2.47 | 2.49 | 0.29 |
| Maha Sarakham | 1-Jun | 25 | 60 | 1.91 | 1.90 | 0.17 |
| Nakhon Ratchasima | 10-Jun | 24 | 61 | 1.78 | 1.82 | 0.12 |
| Nong Bua Lam Phu | 20-Jun | 24 | 60 | 1.87 | 1.87 | 0.14 |
| Phayao | 20-Jun | 30 | 51 | 2.94 | 2.99 | 0.22 |
| Sa Kaeo | 10-Jun | 25 | 62 | 1.78 | 1.82 | 0.24 |

Table S7. Calibration parameters (planting date, harvest index, and fertilizer stress) used in the AquaCrop simulation and the average simulated rice yields for the Vietnam provinces. RMSE: Root mean square error (Calibration period: 1995 – 2004)

|  | Planting date | Harvest index (%) | Fertilizer stress (%) | Observed average yield (t/ha) | Simulated average yield (t/ha) | RMSE (ton/ha) |
| --- | --- | --- | --- | --- | --- | --- |
| Dac Nong | 20-Apr | 35 | 22 | 5.06 | 5.03 | 0.60 |
| Dak Lak | 20-Apr | 36 | 22 | 5.06 | 5.06 | 0.60 |
| Gia Lai | 20-Apr | 37 | 22 | 5.28 | 5.27 | 0.28 |
| Kon Tum | 20-Apr | 33 | 23 | 4.54 | 4.55 | 0.39 |
| Lam Dong | 20-Apr | 29 | 30 | 5.21 | 3.85 | 0.48 |
| An Giang | 20-Jun | 33 | 29 | 4.42 | 4.46 | 0.61 |
| Bac Lieu | 20-Jun | 33 | 29 | 4.46 | 4.51 | 0.49 |
| Ben Tre | 1-Jul | 30 | 30 | 4.10 | 4.09 | 0.16 |
| Ca Mau | 20-Jun | 31 | 30 | 4.23 | 4.23 | 0.48 |
| Can Tho | 1-Jul | 30 | 30 | 4.05 | 4.04 | 0.29 |
| Dong Thap | 20-Jun | 31 | 30 | 4.15 | 4.17 | 0.33 |
| Kien Giang | 1-Jul | 30 | 31 | 3.98 | 3.95 | 0.41 |
| Soc Trang | 1-Jul | 34 | 29 | 4.65 | 4.66 | 0.45 |
| Tra Vinh | 1-Jul | 31 | 30 | 4.29 | 4.26 | 0.39 |
| Vinh Long | 1-Jul | 30 | 30 | 4.06 | 4.06 | 0.18 |

**References**

Abbaspour, K.C., 2011. User Manual for SWAT-CUP: SWAT Calibration and Uncertainty Analysis Programs. Eawag: Swiss Fed. Inst. of Aquat. Sci. and Technol., Duebendorf, Switzerland, p.103.

Hao, Z. and AghaKouchak, A., 2014. A nonparametric multivariate multi-index drought monitoring framework. Journal of Hydrometeorology, 15(1), pp.89-101.

Mainuddin, M., Kirby, M. and Hoanh, C.T., 2012. Water productivity responses and adaptation to climate change in the lower Mekong basin. Water international, 37(1), pp.53-74.

Mainuddin, M., Kirby, M. and Hoanh, C.T., 2013. Impact of climate change on rainfed rice and options for adaptation in the lower Mekong Basin. Natural hazards, 66(2), pp.905-938.

Moriasi, D.N., Arnold, J.G., Van Liew, M.W., Bingner, R.L., Harmel, R.D. and Veith, T.L., 2007. Model evaluation guidelines for systematic quantification of accuracy in watershed simulations. Transactions of the ASABE, 50(3), pp.885-900.

Palmer, W.C., 1965. Meteorological drought (Vol. 30). US Department of Commerce, Weather Bureau.

Sridhar, V., Kang, H. and Ali, S.A., 2019. Human-induced alterations to land use and climate and their responses for hydrology and water management in the Mekong River Basin. Water, 11(6), p.1307.
